# Nerve Growth Factor is Sufficient to Cause Multiple Osteoarthritis‐Relevant Pathologic Features in Naïve Murine Knee Joints

**DOI:** 10.1002/art.70168

**Published:** 2026-05-10

**Authors:** Alia M. Obeidat, Michael D. Newton, Jun Li, Baofeng Hu, Shingo Ishihara, Lindsey Lammlin, Lucas M. Junginger, Easton C. Farrell, Lai Wang, Frank C. Ko, Richard J. Miller, Carla R. Scanzello, Tristan Maerz, Rachel E. Miller, Anne‐Marie Malfait

**Affiliations:** ^1^ Department of Internal Medicine, Division of Rheumatology Rush University Medical Center Chicago Illinois; ^2^ Chicago Center on Musculoskeletal Pain (C‐COMP) Chicago Illinois; ^3^ Department of Orthopaedic Surgery University of Michigan Ann Arbor; ^4^ Department of Medicine, Division of Rheumatology University of Pennsylvania Philadelphia; ^5^ Translational Musculoskeletal Research Center Corp. Michael J. Crescenz VA Medical Center Philadelphia Pennsylvania; ^6^ Institute for Biomechanics, Department of Health Sciences and Technology ETH Zürich Zürich Switzerland; ^7^ Department of Anatomy & Cell Biology Rush University Medical Center Chicago Illinois; ^8^ Department of Pharmacology Northwestern University Feinberg School of Medicine Chicago Illinois

## Abstract

**Objective:**

Nerve growth factor (NGF), a key mediator of pain, is increased in osteoarthritic (OA) joints. Antibodies against NGF show analgesic effects in painful knee OA, but clinical development was stopped because of side effects in the joints. Knowledge about the biologic effects of NGF on joint tissues is limited. Therefore, we explored the effects of repeated intra‐articular (IA) injections of NGF into naïve murine knee joints on sensitization, joint innervation, and histopathology.

**Methods:**

Naïve 10‐ to 15‐week‐old male wildtype C57BL/6 mice were injected with NGF (50 or 500 ng) or vehicle IA twice a week for four weeks, and effects on knee swelling, knee hyperalgesia, joint histopathology, and bone were assessed. Single cell RNA sequencing (scRNAseq) of the synovium was performed. Na_V_1.8‐tdTomato reporter mice were used to assess joint innervation. Dorsal root ganglia (DRGs) of mice underwent bulk RNAseq after three IA injections of NGF or vehicle.

**Results:**

Compared with vehicle, repeated IA injections of NGF caused dose‐dependent increases in knee swelling, knee hyperalgesia, synovial pathology, bone mineral density in the medial subchondral bone, and medial preosteophytes, but no overt cartilage damage. NGF caused increased sprouting of nociceptors in the medial synovium that was preceded by up‐regulation of axonal growth pathways in the DRGs. ScRNAseq of the synovium revealed up‐regulated genes related to neuronal sprouting, synovial fibrosis, and ossification, with a key role for lining fibroblasts.

**Conclusion:**

In naïve mouse knees, NGF induced many pathologic changes observed in OA, including nociceptor sprouting, suggesting a critical role for NGF in OA pathogenesis.

## INTRODUCTION

The neurotrophin, nerve growth factor (NGF), is essential for the growth and survival of sensory and sympathetic neurons during embryonic development.^1^ In adulthood, NGF is produced at low basal levels but is rapidly up‐regulated during inflammation, acting as a key mediator of neuroimmune interactions in inflammatory processes and pain.^2^ NGF can be produced by many cell types, including epithelial and endothelial cells, fibroblasts, smooth muscle cells, glial cells, and immune cells.^2^ NGF exerts its effects through binding to two classes of receptors: the high affinity receptor tyrosine receptor kinase A (TrkA) and the low affinity p75 neurotrophin receptor (p75^NTR^). Both receptors are broadly expressed and involved in neuronal development and pain sensitization.^2^, ^3^

NGF is associated with several painful inflammatory conditions^2^, ^3^, ^4^ and has received extensive attention as a target for pain in osteoarthritis (OA).^5^ OA is the most common form of arthritis, and with an estimated 600 million people globally suffering from symptomatic OA,^6^ it is one of the major sources of chronic pain in the world. Current pain management options include conventional analgesics such as nonsteroidal anti‐inflammatory drugs (NSAIDs) and intra‐articular (IA) strategies such as glucocorticoids, but these show limited efficacy or are associated with safety concerns after prolonged use.^7^ Clinical trials with neutralizing anti‐NGF antibodies showed considerable efficacy in treating OA pain, but drug development programs were ceased after the US Food and Drug Administration failed to approve anti‐NGF antibodies in 2021 because of the risk of rapidly progressive OA (RPOA).^5^ The mechanisms underlying this adverse effect are poorly understood, but the observation that NGF blockade may affect joint integrity raises questions as to the biologic functions of NGF in the joint. Both NGF receptors are widely distributed in joint tissues: TrkA is not only expressed by nociceptors, but also by immune cells (macrophages, T cells, mast cells), chondrocytes, fibroblasts and bone forming cells.^2^, ^4^, ^8^ p75^NTR^ is expressed by chondrocytes, fibroblasts, immune cells, and Schwann cells.^2^, ^3^

NGF protein levels are elevated in OA synovial fluid, as in rheumatoid arthritis and spondyloarthritis^9^, ^10^ and in the serum of individuals with OA.^10^ In joints of people or laboratory animals with OA, NGF messenger RNA (mRNA) is increased in chondrocytes,^11^, ^12^, ^13^ synovial fibroblasts, and macrophages.^14^, ^15^ The sensitizing and pain‐producing effects of IA NGF injections into rodent knee joints have been well documented. A single IA injection into healthy rat knees induced weight‐bearing asymmetry, mechanical allodynia, and knee swelling with synovial macrophage infiltration.^16^ In naïve mice, repeated NGF injections resulted in knee swelling and knee hyperalgesia.^17^ In contrast, little is known about the effects of NGF on different joint tissues, especially not in a whole‐joint context. To address this gap in knowledge, we assessed the biologic actions of NGF in the joint by injecting NGF into the knees of naïve, young adult mice. We assessed knee swelling and hyperalgesia, histopathology and innervation of joint tissues, and molecular architecture of the synovium and innervating dorsal root ganglia (DRG) to gain insight into how NGF may regulate processes involved in joint damage and pain.

## MATERIALS AND METHODS

### Animals

We used a total of 65 male C57BL/6 mice that were either wildtype (WT) (inbred at Rush) or Na_V_1.8‐tdTomato mice (which express a tdTomato reporter in >90% of nociceptors).^18^ Animals were housed with food and water ad libitum and kept on a 12‐hour light cycle. All animal procedures were approved by the Rush Institutional Animal Care and Use Committee.

### Injection protocols and experimental designs

All injection protocols are depicted in Supplemental Figure 1. Injection regimen and time of delivery were determined based on pilot studies in which multiple conditions were tested. The amount of NGF used in the study is higher than that typically found in the osteoarthritic joint.^19^ This was required to ensure sufficient concentration of NGF is in the joint and to overcome the initial clearance of the protein following each injection.^20^ In experiment 1, NGF (R&D systems, # 1156‐NG‐100), 50 ng or 500 ng in 5 μL, or vehicle control (0.1% BSA) (05470, Sigma) (n = 5 mice/group) were injected IA into the right knee of naïve 12‐week‐old male WT mice twice a week for four weeks. These mice were used to assess knee swelling, knee hyperalgesia, and histopathology. In experiment 2, a cohort of WT mice was used to assess the tibial subchondral bone using microcomputed tomography (μCT) (n = 4 vehicle and n = 6 NGF, 500 ng). This cohort had been used in a previous study.^21^ In experiment 3, NGF (500 ng) or vehicle were injected into the right knee of 12‐week‐old male Na_V_1.8‐tdTomato mice (n = 5 mice/group) two times a week for four weeks. These mice were used for innervation studies. In experiment 4, 12‐week‐old WT mice received NGF (500 ng) or vehicle two times a week for four weeks (n = 9/group), and knee hyperalgesia was assessed. After four weeks, synovium was collected for single cell RNA sequencing (scRNAseq) from four randomly selected mice. Last, in experiment 5, 15‐week‐old WT mice received NGF (500 ng) or vehicle twice a week for 10 days (3 IA injections total, n = 6/group). Twenty‐four hours after the last injection, L3 to L5 ipsilateral DRGs were collected for bulk RNAseq.

### Knee swelling and knee hyperalgesia

In experiment 1, knee width was measured at baseline (before the first injection) and then twice a week, right before each injection, using microcalipers. Knee hyperalgesia was assessed at baseline and then at weeks two and four by an observer blinded to treatment groups (SI), using a pressure application measurement device as detailed in the Supplemental Methods.

### Knee histopathology

Mice in experiment 1 were euthanized after four weeks (age = 16 weeks), and the right knees were used for evaluation of cartilage damage using modified Osteoarthritis Research Society International recommendations^22^ (see Supplemental Methods). For osteophyte scoring, one section with the major chondrophyte or preosteophyte was assessed using Osteomeasure software (OsteoMetrics).^23^ Synovial pathology (hyperplasia, cellularity, and fibrosis) was evaluated in four joint spaces (lateral‐femoral, medial‐femoral, lateral‐tibial, and medial‐tibial), as described.^24^ Five 5‐μm sections per mouse were assessed, two from anterior to midpoint, a midpoint section, and two from the posterior area. Scoring was performed by four observers blinded to the groups (AMO, JL, BH, CRS). Scores were averaged for all five sections per area per mouse for each scorer. Scores from all four observers were averaged and graphed per parameter.

### μCT

μCT imaging was performed on WT mice that were injected with either vehicle (n = 4) or NGF (500 ng, n = 6) using a high‐resolution laboratory imaging system (μCT50, Scanco Medical AG) in accordance with the American Society of Bone and Mineral Research guidelines for the use of μCT in rodents.^25^ We evaluated the bone volume/trabecular volume fraction (mm^3^/mm^3^), bone mineral density (BMD) (mg HA/cm^3^), mean trabecular thickness (Tb.Th, mm), and mean trabecular separation (Tb.Sp, mm). Representative image of the region of interest is shown in Supplemental Figure 2. Details are in the Supplemental Methods.

### Quantification of the neuronal signal in the synovium

Na_V_1.8‐tdTomato mice were perfused with 4% paraformaldehyde at week 4, and right knees were collected. Coronal sections of 20‐μm thickness were collected from the mid‐joint area, as defined in Obeidat et al.^26^ Sections were imaged using a laser‐scanning confocal microscope (Olympus IX70) and processed using ImageJ and Fluoview software (FV10‐ASW 4.2 Viewer). Details are in the Supplemental Methods.

### ScRNAseq of the synovium

Methods for synovial scRNAseq are detailed in the Supplemental Methods. Briefly, 8 C57Bl/6 mice (n = 4/condition) were randomized to four weeks of biweekly NGF (500 ng) or vehicle injections. Mice were euthanized via CO_2_ asphyxia, and ipsilateral synovia (including medial and lateral anterior synovium, along with Hoffa's fat pad) were collected and digested to yield a single cell suspension^27^ that was frozen in liquid nitrogen and submitted to the University of Michigan Advanced Genomics Core for 10X Genomics scRNAseq processing (Chromium Next GEM Single Cell 3′ Kit v3.1). Two bioreplicates were submitted per condition, with each bioreplicate pooled from two synovia. ScRNAseq quality control details (sequencing accuracy control, cell quality metrics before and after preprocessing) are provided in the Supplemental Methods, Supplemental Figure 3A and B, and Supplemental Table 1. Samples were integrated and clustered to identify unique cell populations. Clusters were annotated by expression of unique, cell type‐defining gene markers. Spearman correlation confirmed high correlation between bioreps within each condition (Supplemental Figure 3C). Differential gene expression (DEG) analysis, along with functional annotation via gene set enrichment analysis (GSEA) was performed within each cell type between NGF and vehicle groups.^28^ *P* values for DEG results were computed using the Wilcoxon Rank Sum test, corrected for multiple comparisons via the Bonferroni correction, and significant DEGs were defined as Padj <0.05, |log2(fold change)| > 0.585. GSEA results were corrected for multiple comparisons via the Benjamini–Hochberg correction, and significant terms were defined as Padj <0.05. To determine which transcription factors were putatively underpinning expression of NGF‐induced changes, regulon analysis was performed via RcisTarget.^28^

### Bulk RNAseq of lumbar DRGs

Ipsilateral DRGs (L3–L5) were collected from male WT mice after 3 IA injections of 500 ng NGF (n = 6) or vehicle (n = 6) over 10 days. In contrast to all other experiments, we used 15‐week old male mice (instead of 12 weeks) to age match the mice from our previous study.^17^ At takedown, these mice were close to the age of takedown in the other experiments (ie, 16 weeks). Because NGF is retrogradely transported to the DRG and induces transcriptional changes,^3^ we sought to describe molecular changes in the DRG at an early time point, when mice show knee hyperalgesia but preceding joint innervation changes. Therefore, we injected 500 ng/mL NGF just three times over the course of 10 days and then harvested the ipsilateral DRGs. DRGs were collected 24 hours after the last injection, and RNA was extracted using an RNeasy micro kit (Qiagen) and sent to LC Sciences for sequencing. The raw sequence data and counts matrixes were submitted to NCBI GEO with accession number GSE287526. mRNA DEG analysis was performed by R package DESeq2^29^ between two different groups (NGF vs vehicle). For DRG Bulk RNAseq quality control details, please refer to Supplemental Table 2. Details are in the Supplemental Methods.

### Statistical analysis

Sample size was determined based on previous data comparing innervation changes between sham and destabilization of the medial meniscus (DMM).^26^ Ordinary one‐way analysis of variance (ANOVA) followed by Sidak's post‐test was used for multiple group comparisons of synovial fibrosis and cartilage degeneration. The Kruskal–Wallis test with Dunn's multiple comparisons was used for total knee hyperplasia, cellularity, and osteophyte width. Ordinary two‐way ANOVA followed by Dunnett's post‐test was used for multiple group comparisons of the knee hyperalgesia time course. Unpaired two‐tailed Student's *t*‐test was applied for comparison of the lateral synovial innervation and bone morphometric analyses (except for lateral mean trabecular separation) between two groups. The Mann–Whitney test was used to compare two groups for medial synovium innervation, medial channels, knee swelling, and knee hyperalgesia of the scRNA‐seq experiment. For one‐way and two‐way ANOVAs, pair‐wise tests were only performed if the overall *P* value was <0.05. The resulting pair‐wise test *P* values are reported. Statistical analyses were performed using GraphPad Prism 9. Data are presented as mean ± SEM.

## RESULTS

### IA administration of NGF causes synovitis accompanied by knee swelling and knee hyperalgesia

We first sought to assess the effects of repeated IA injections of NGF on the development of synovitis, knee swelling, and knee hyperalgesia (experiment 1). We confirmed previous findings that repeated IA administration of NGF causes knee swelling.^17^ The high dose (8 × 500 ng) induced knee swelling as early as four days after the first injection, with a sustained effect up to 31 days. The low dose (8 × 50 ng) did not induce knee swelling (*P* = 0.3011) (Supplemental Figure 4A and B). All mice developed knee hyperalgesia after IA injections and after vehicle administration (Supplemental Figure 4C). However, mice that received NGF, 50 ng or 500 ng, developed significantly more knee hyperalgesia after two weeks compared with the vehicle group. By week four, knee hyperalgesia was more pronounced in the high‐dose NGF group compared with the vehicle group but had partially subsided in the low‐dose group without returning to baseline levels (Supplemental Figure 4C). No significant differences were detected between the two NGF groups at either time point (*P* = 0.9444 at week 2 and *P* = 0.1325 at week 4). Detailed description of all *P* values is in Supplemental Table 3.

NGF had striking effects on synovial histopathology (Figure 1), with the high dose causing marked changes in the medial and the lateral synovium, with increased subintimal cellularity, fibrosis, and lining hyperplasia (Figure 1A–C). Effects of low‐dose NGF were less pronounced, with increased cellularity in the medial synovium (Figure 1A) and a slight increase in fibrosis and hyperplasia that was not significant compared with controls (*P* = 0.0660 and *P* = 0.0856, respectively) (Figure 1B and C). We observed no differences between the low NGF and vehicle groups in the lateral synovium (Figure 1A–C). Representative histologic images of the synovium are shown in Figure 1D–F.

**Figure 1:**
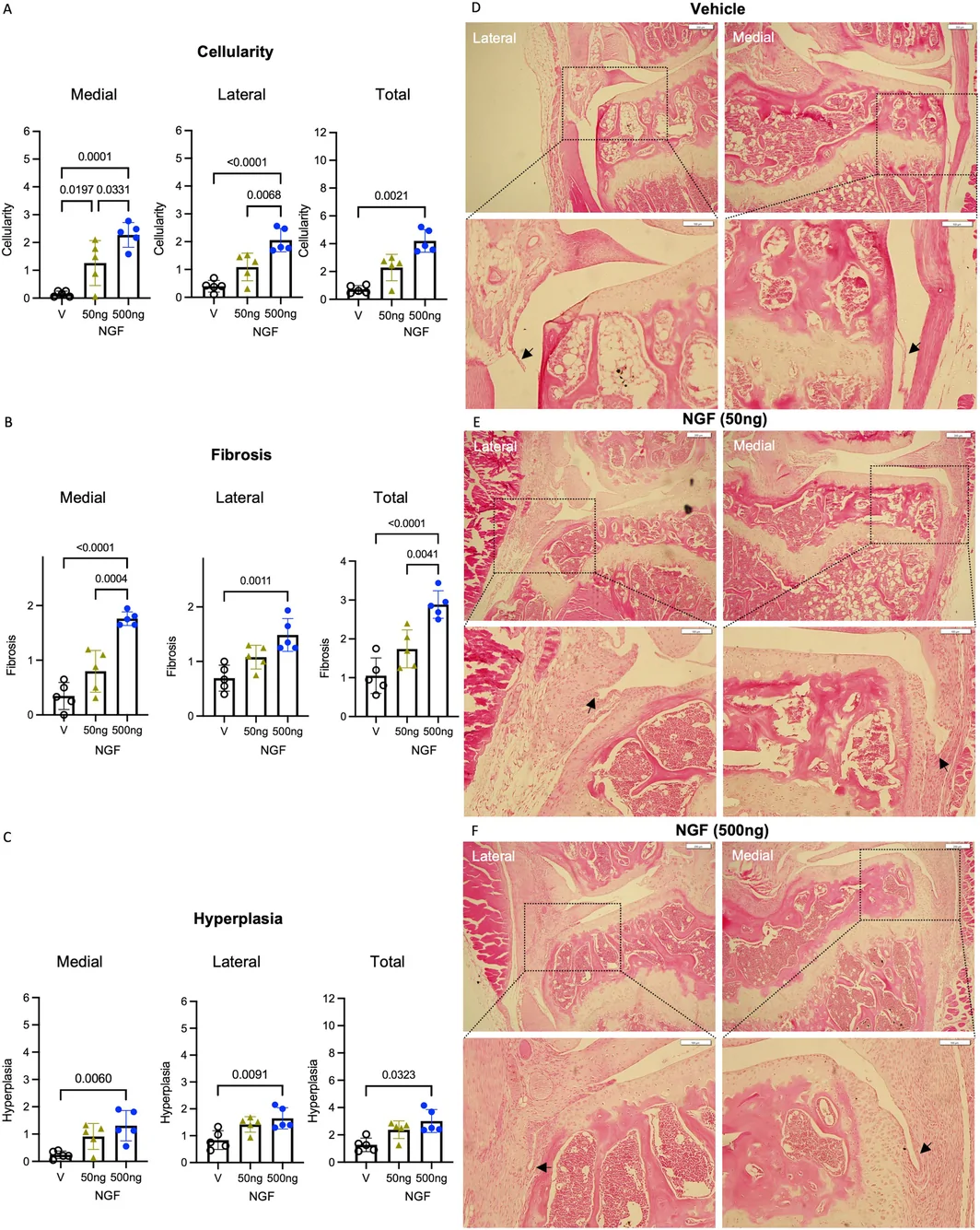
(A) Synovial cellularity, (B) fibrosis, and (C) hyperplasia were evaluated in WT mice that received repeated intra‐articular injections of recombinant murine NGF (IA 50 ng or 500 ng, 5 μL, 2×/week) or vehicle (5 μL, 2×/week) for four weeks; n = 5 mice/group. Scores are shown for the medial and the lateral compartments, as well as the total joint scores. Representative histologic images of medial and lateral tibial synovia showing overview and zoomed‐in images (in black insets) from the knee sections of mice injected with (D) vehicle, (E) 50 ng NGF, or (F) 500 ng NGF mice, respectively. Histologic images show all three pathologic features scored in (A)–(C). The zoomed in images in black insets show fibrosis and increased cellularity in NGF‐injected mice. Black arrows show representative areas used to score hyperplasia. For (A)–(C), we used one‐way ANOVA with Sidak's post‐test or Kruskal‐Wallis test with Dunn's Multiple Comparisons if these data are nonparametric. Scale bar = 200 μm for overview images and 100 μm for zoomed‐in images. ANOVA, analysis of variance; IA, intra‐articular; NGF, nerve growth factor; WT, wildtype.

### IA administration of NGF causes subchondral bone changes but not cartilage damage

Chondrocytes express both NGF receptors, and OA cartilage exhibits increased NGF mRNA.^11^ We did not observe overt cartilage degeneration in any of the experimental groups after eight injections (Figure 2A). Articular surfaces were intact, with no evidence of surface fibrillation. However, in the medial compartment, small chondrophytes or preosteophytes developed in response to NGF in a dose‐dependent manner (Figure 2B). Representative histologic images are shown in Figure 2B and Supplemental Figure 5. Moreover, μCT analysis revealed that eight injections of 500 ng NGF led to bone changes in the medial‐tibial subchondral bone, for which BMD and trabecular bone thickness were significantly increased compared with controls, whereas no differences in bone volume fraction or mean trabecular spacing were observed (Figure 2C–F). No changes in bone morphometric parameters were observed in the subchondral bone of the lateral compartment (Figure 2C–F). Representative 3D reconstructions of the subchondral trabecular bone from NGF‐treated mice demonstrated a sclerotic morphology reminiscent of OA (Figure 2G).

**Figure 2:**
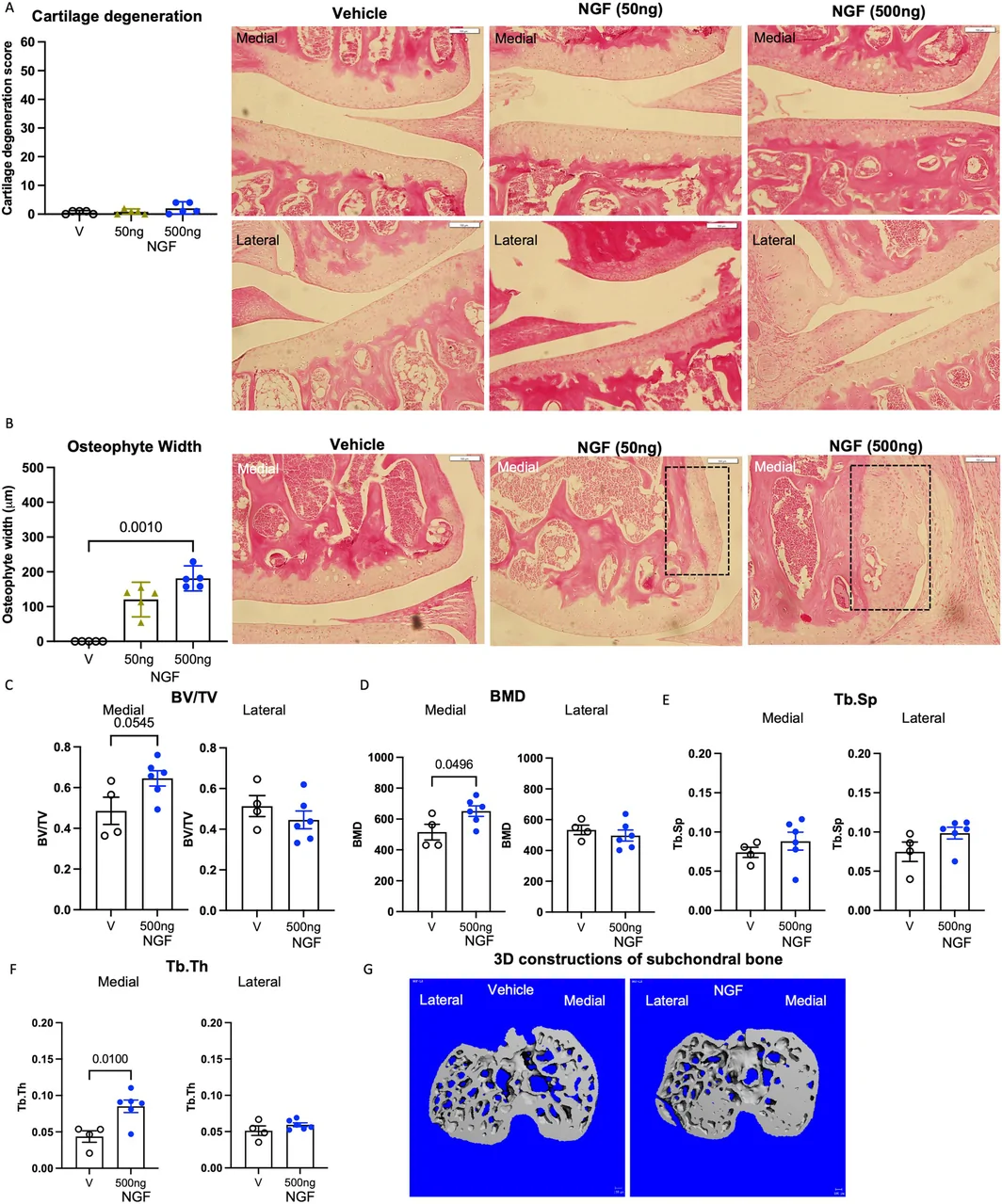
(A) Cartilage degeneration scores and representative histologic images of knee sections from WT mice injected with vehicle, low NGF, or high NGF, respectively. (B) Small preosteophytes developed on the medial side. The graph shows the width in μm with representative histologic images of WT mice injected with vehicle (5 μL, 2×/week), low NGF (50 ng, 5 μL, 2×/week), or high NGF (500 ng, 5 μL, 2×/week) for four weeks, n = 5 mice/group. Black insets in (B) showing examples of developing preosteophytes in low dose and high dose NGF groups. (C) BV/TV ratio, (D) bone mineral density (BMD, in mm), (E) mean trabecular thickness (Tb.Th, in mm), and (F) mean trabecular separation (Tb.Sp, in mm), respectively, in the medial and lateral tibial subchondral trabecular bone from knees injected with NGF (500 ng, n = 6) or vehicle (n = 4). (G) Representative μCT scans of the subchondral trabecular bone from vehicle and NGF groups, respectively. For (A), we used one‐way ANOVA with Sidak's post‐test; For (B), Kruskal–Wallis test with Dunn's Multiple Comparisons Test; For (C)–(F), we used an unpaired two‐tailed *t*‐test, or if the data were nonparametric, a Mann–Whitney test was used (E) (lateral). Scale bar = 100 μm. ANOVA, analysis of variance; BV/TV, trabecular bone volume fraction; NGF, nerve growth factor; μCT, microcomputed tomography; WT, wildtype.

### IA injection of NGF causes nociceptor sprouting in the medial synovium and into preosteophytes

Nociceptor sprouting was observed in subchondral bone and synovium during experimental OA.^23^, ^26^ To determine whether NGF alone is sufficient to induce this process thought to underpin pain in OA, we evaluated the Na_V_1.8+ nociceptive knee innervation after eight injections of 500 ng NGF or vehicle. In the medial synovium, we observed no or very few nociceptors in the vehicle‐injected group, in line with previous reports,^26^ whereas NGF injection caused sprouting of Na_V_1.8+ fibers in the deep layers (Figure 3A–C). Representative confocal images of the medial synovium are shown in Figure 3B and C. The lateral synovium of naïve 16‐week old mice contained more nociceptors than the medial synovium, as reported before,^26^ and no changes were detected among groups (Figure 3D) (representative images of the lateral synovium in Figure 3E and F). We did not detect changes in the innervation pattern of the subchondral bone between groups (Supplemental Figure 6A–C), but we observed nociceptors sprouting into growing preosteophytes (Supplemental Figure 6D). The signal within the preosteophytes has previously been confirmed to be neuronal.^23^, ^26^

**Figure 3:**
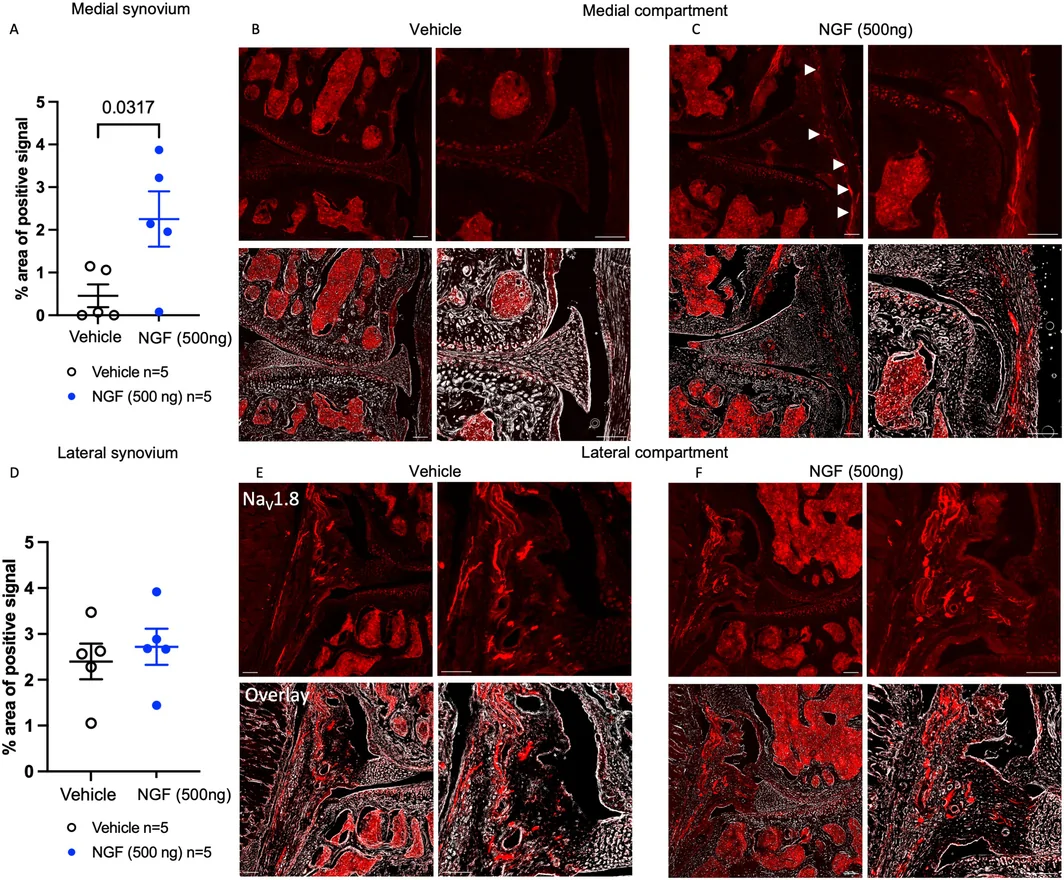
(A) Quantification of neuronal signal in the medial synovium of Na_V_1.8‐tdTomato mice injected with vehicle (5 μL, 2×/week) or recombinant murine NGF (IA 500 ng, 5 μL, 2×/week) for four weeks; n = 5 mice/group. Representative confocal images with zoomed‐in images of the (B) medial compartment of vehicle and (C) NGF‐treated groups showing Na_V_1.8 fibers in white arrows. (D) Quantification of neuronal signal in the lateral synovium of Na_V_1.8‐tdTomato mice injected with vehicle (5 μL, 2×/week) or recombinant murine NGF (500 ng, 5 μL, 2×/week) for four weeks; n = 5 mice/group. Representative confocal images of the (E) lateral compartment of vehicle and (F) NGF‐treated groups. We used an unpaired two‐tailed *t*‐test or a Mann−Whitney test if data are nonparametric. Scale bar = 100 μm. IA, intra‐articular; NGF, nerve growth factor.

### ScRNAseq of the synovium

Given the profound pathologic changes in synovium following NGF administration, we performed scRNAseq on synovium from mice injected IA with NGF or vehicle to assess the cell type‐specific transcriptional signatures underpinning these effects. Consistent with experiment 1, mice developed knee hyperalgesia after four weeks of high‐dose NGF (Supplemental Figure 7). Following quality control and preprocessing, 17,041 high‐quality cells were identified (vehicle: n = 8,170, NGF: n = 8,871) (Supplemental Figure 8A). Twelve major transcriptionally‐distinct cell type clusters were identified and annotated (Figure 4A, Supplemental Figure 8E), expressing canonical, lineage‐specific cell markers (Figure 4C, Supplemental Figure 8D) consistent with our experience in mouse synovium.^27^ NGF‐treated samples had marginally higher sublining fibroblast content (Figure 4B) consistent with our observation of synovial fibrosis (Figure 1A–C). Other cell types did not vary appreciably in abundance by group, and no emergence of new transcriptionally‐distinct clusters was observed.

**Figure 4:**
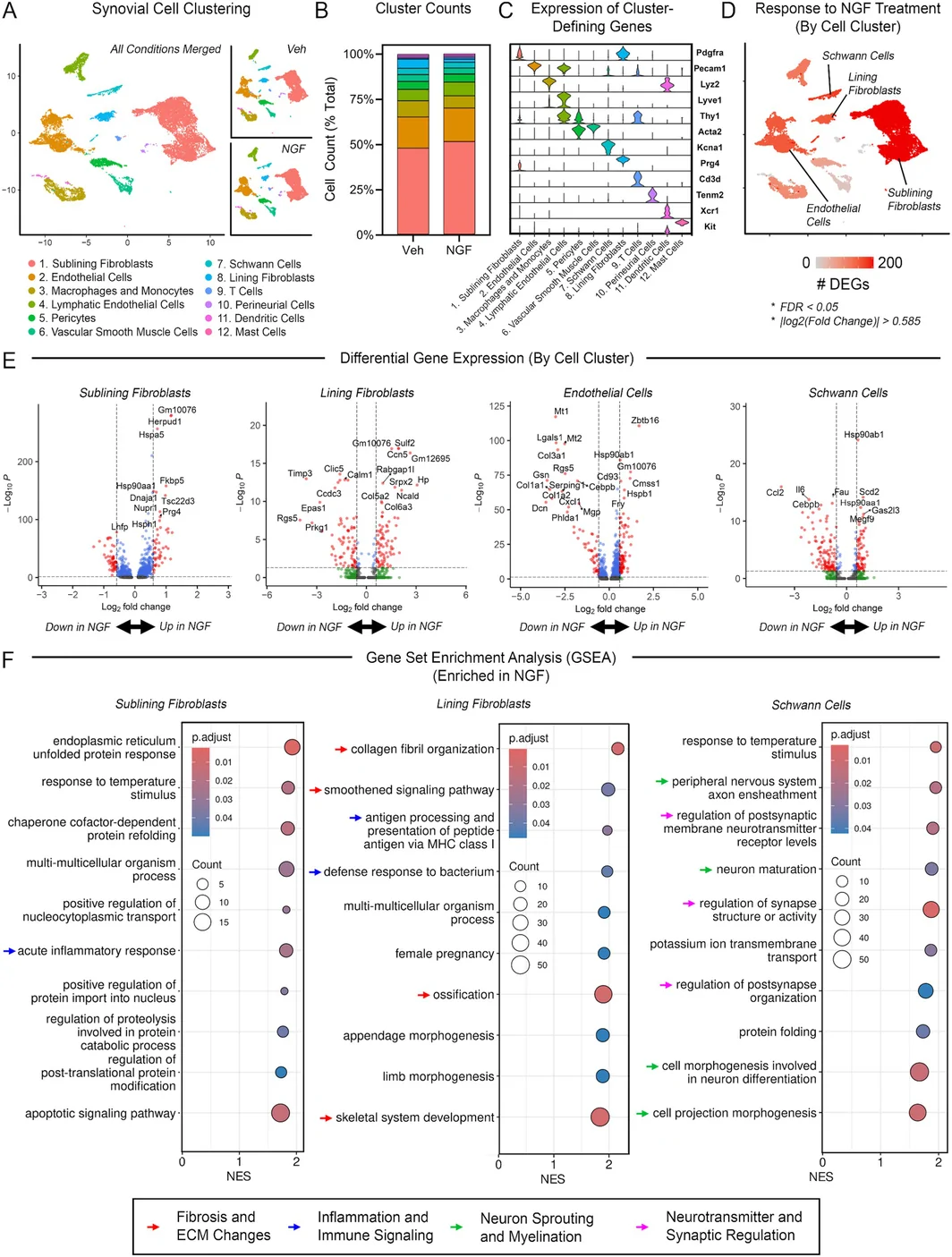
Single cell RNAseq of whole synovium response to IA NGF injection. (A) Annotation of distinct synovial cell clusters. Twelve major cell types were identified, and annotated fibroblasts (sublining and lining), endothelial cells (vascular and lymphatic), and macrophages/monocytes comprised the majority; smaller populations of pericytes, VSMCs, Schwann cells, T cells, perineurial cells, and dendritic cells were also detected. (B) Proportional cell counts of synovial cells in vehicle and NGF groups were similar. (C) Expression of cluster‐defining genes. (D) Cluster‐specific response to NGF treatment, enumerated as number of DEGs per cluster, shows the largest number of NGF‐induced differences in gene expression that occurred in sublining and lining fibroblasts, endothelial cells, and Schwann cells. NGF‐induced differential gene expression of these clusters is shown as (E) volcano plots of differential gene expression (cutoffs at *P*_*adj*_ < 0.05 and |log2(fold change)| > 0.585) and (F) gene set enrichment (top 10 enriched pathways shown, sorted by NES). DEG, differentially expressed gene; FDR, false dicsovery rate; IA, intra‐articular; MHC, major histocompatibility complex; NES, normalized enrichment score; NGF, nerve growth factor; VSMC, vascular smooth muscle cell.

Differential‐expression and gene set enrichment analyses demonstrated robust, multifaceted NGF‐induced transcriptional responses across several cell types, lining fibroblasts (154 DEGs), sublining fibroblasts (102 DEGs), vascular endothelial cells (154 DEGs), and Schwann cells (180 DEGs) (*P*_*adj*_ < 0.05, |log_2_(fold change)|>0.585) (Figure 4D and E, Supplemental Figures 9 and 10). Vascular endothelial cells were enriched in pathways consistent with structural changes observed in NGF‐treated knees (Figure 4F). Schwann cells were enriched in pathways related to neuronal growth and myelination, with up‐regulation of *Megf9* and *Gas2I3*, and up‐regulated heat shock protein pathway genes (*Hsp90aa1*, *Hsp90ab1*) postulated to play a role in neuron development and regulation. Broad heat shock protein pathway activation was observed in sublining fibroblasts, vascular and lymphatic endothelial cells, macrophages and monocytes, pericytes, and vascular smooth muscle cells, suggesting NGF may activate a generalized stress response. Sublining and lining fibroblasts exhibited enrichment of pro‐inflammatory pathways (Figure 4F), with lining fibroblasts enriched in pathways related to innate immunity, marked by up‐regulation of *Hp* (haptoglobin) and histocompatibility genes *H2‐D1*, *H2‐K1*, and *H2‐T22*.

Lining fibroblasts in particular exhibited NGF‐induced enrichment of several pathways reflective of synovial histopathology, including fibrosis (collagen fibril organization, smoothened signaling pathway) and osteochondral bone formation (ossification, skeletal system development) (Figure 4F). At the gene‐level, lining fibroblasts up‐regulated numerous genes related to fibrosis and endochondral ossification: collagens, including fibrosis‐associated *Col3a1*, *Col5a1*, and *Col6a1*; crosslinking enzymes (*Lox*, *Loxl1*); linking glycoproteins (eg, *Emilin1*); fibrosis‐ and endochondral ossification‐associated signaling pathways, including Wnt (*Rspo2*, *Serpinh1*) and Smoothened (*Smo*, *Gli3*, *Gas1*); transcriptional regulators known to drive extracellular matrix target genes (*Runx1*, *Tgfbr3*, *Egr2*); and matrix‐associated factors and proteases (*Thbs3*, *Ccn1*, *Mmp2*) related to osteogenesis (Figure 5A). For detailed references on all these pathways, please see the Supplemental Results. To interrogate putative transcriptional mediators underlying these changes, we performed a regulon analysis of all significant DEGs up‐regulated by lining fibroblasts. This analysis predicted one significantly enriched, directly‐annotated binding motif (Figure 5B) that was shared among the Sox family of transcription factors. Lining fibroblasts expressed four Sox family genes (*Sox4*, *Sox5*, *Sox6*, and *Sox9*), with *Sox5* exhibiting markedly higher expression than other Sox genes (Figure 5C). The *Sox5* gene regulatory network via this binding motif implicates Sox5‐driven expression of genes related to the fibrosis‐ and ossification‐related pathways activated by NGF (Figure 5D). Collectively, these findings suggest a key role for lining fibroblasts in driving the pathologic effects of NGF in synovium, with Sox5 identified as the putative transcriptional mediator underpinning the associated transcriptional program.

**Figure 5:**
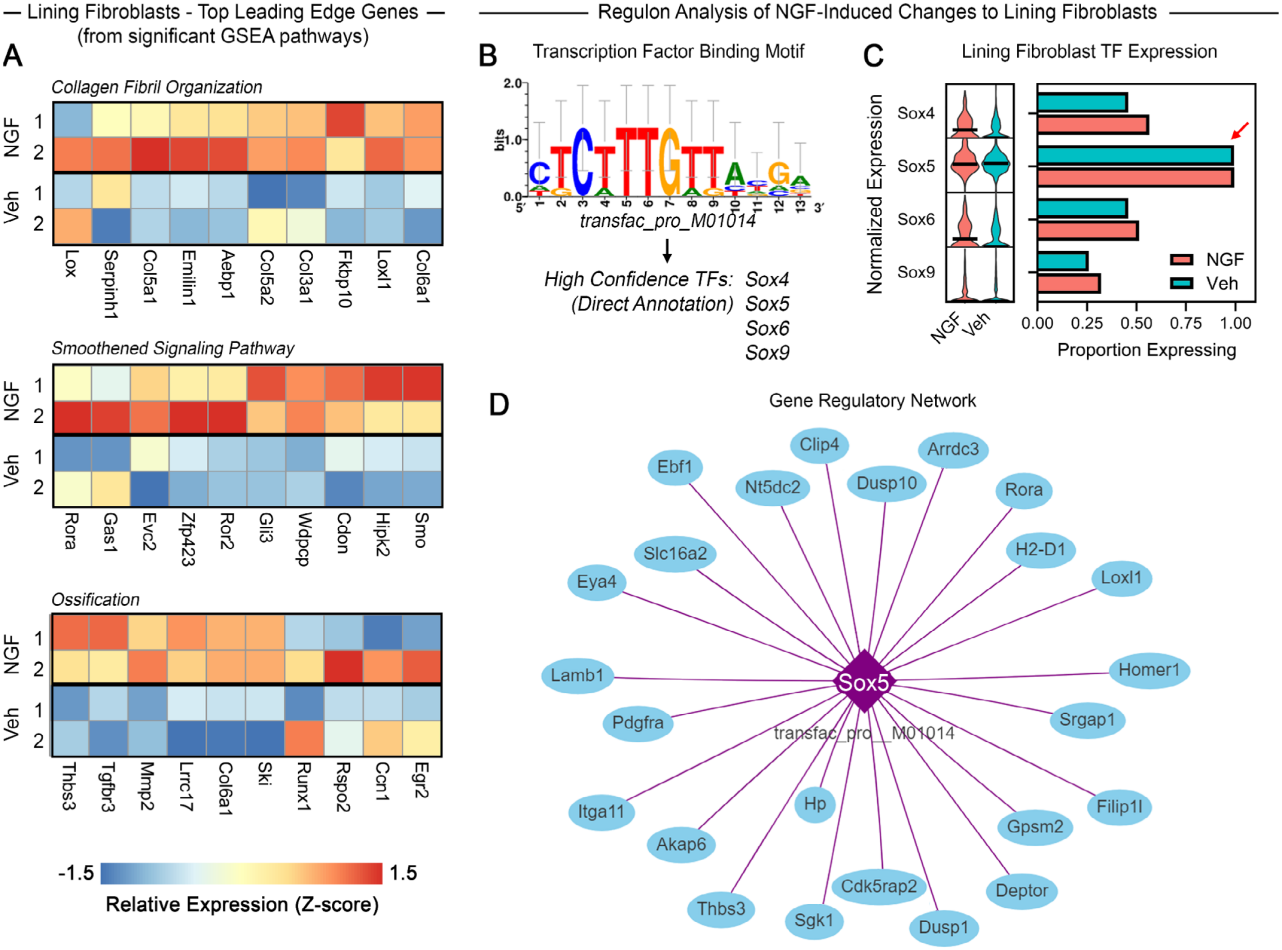
NGF‐induced changes in the lining fibroblast transcriptome. (A) Heatmap of top gene set enrichment analysis‐derived leading‐edge genes, demonstrating coordinated up‐regulation of genes related to collagen fibril organization, smoothened signaling, and ossification. (B) Transcription factor binding motif analysis (RcisTarget) of lining fibroblasts in NGF versus vehicle revealed significantly enriched motif transfac_pre_M01014 for the Sox family of transcription factors, predicted by direct annotation. (C) Expression of predicted transcription factors by lining fibroblasts in vehicle and NGF groups, demonstrating the highest expression of Sox5 (red arrow). (D) Gene regulatory network plot for Sox5 in lining fibroblasts. GSEA, gene set enrichment analysis; NGF, nerve growth factor; TF, transcription factor.

### Bulk RNAseq of the lumbar DRG

Because NGF is retrogradely transported to the DRG and induces transcriptional changes,^3^ we sought to describe molecular changes in the DRG at an early time point, when mice show knee hyperalgesia but preceding joint innervation changes. Bulk RNAseq of ipsilateral L3 to L5 DRGs collected after three IA injections of NGF (500 ng) or vehicle revealed 296 up‐regulated and 415 down‐regulated genes (Figure 6A). The top 50 differentially expressed genes are shown in (Figure 6B), and of these, *Ngfr*, *Cnr1*, *F2r*, *Mmp9*, *Nptx1*, *Npy1r*, and *Ptgir* have been previously linked to pain,^30^ but many had not been previously directly linked to NGF.

**Figure 6:**
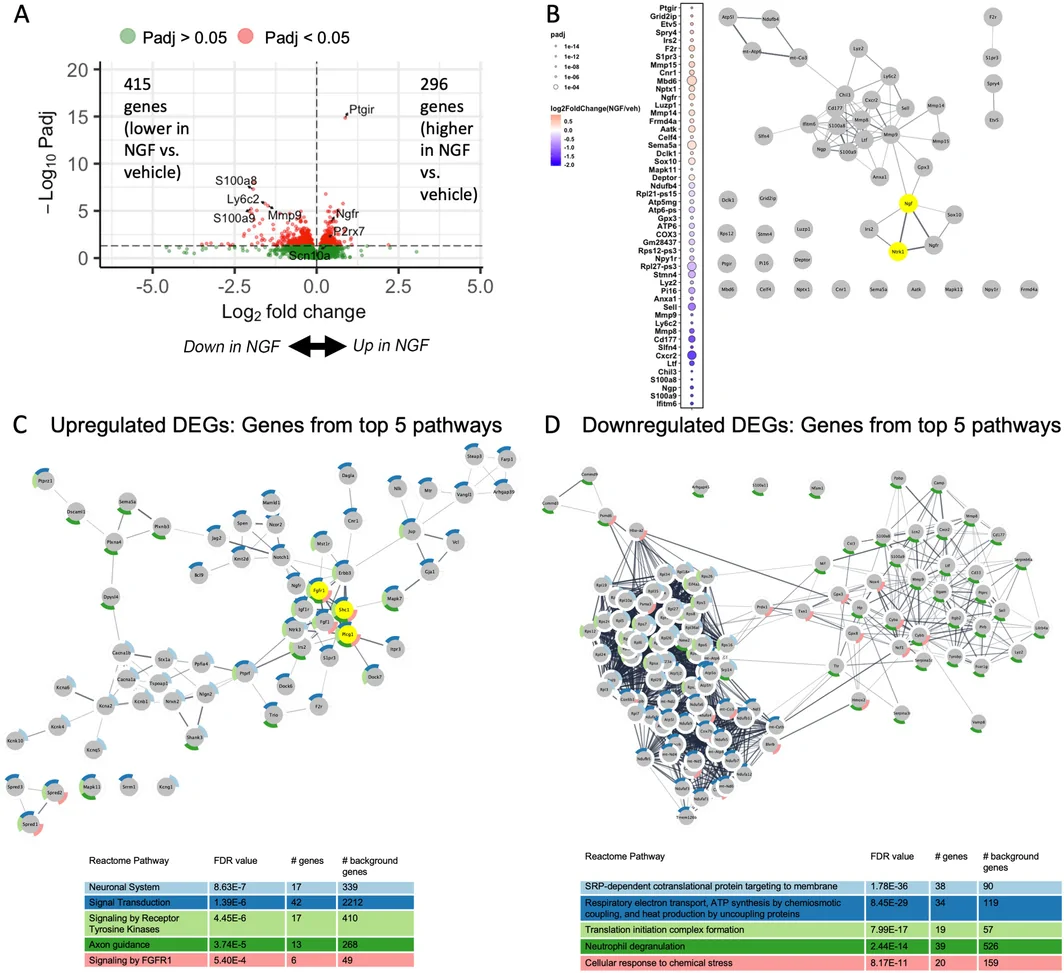
DEG analysis on bulk RNAseq conducted on DRGs collected after three injections of NGF or vehicle. (A) Volcano plot showing all genes that were not filtered out by DESeq2 (15,664). Red indicates genes that met the Padj cutoff <0.05. No fold change cutoff was applied. Genes of interest are labeled. (B) Top 50 genes by Padj, ordered by log2 fold change with the size of the bubble mapped to the adjusted *P* value. String network showing known relationships to Ngf and to Ntrk1 (highlighted in yellow). (C) Functional enrichment was performed on the up‐regulated DEG list using Cytoscape String Enrichment; interacting genes contributing to the top five Reactome pathways are shown using a redundancy cutoff of 0.5. Hub genes are highlighted in yellow. (D) Functional enrichment was performed on the down‐regulated DEG list using Cytoscape String Enrichment; interacting genes contributing to the top five Reactome pathways shown. DEG, differential gene expression; DRG, dorsal root ganglia; FDR, false discovery rate; NGF, nerve growth factor; RNAseq, RNA sequencing.

Functional enrichment analysis of the up‐regulated genes showed that the top five enriched pathways were related to neuroplasticity: neuronal system, signal transduction, signaling by receptor tyrosine kinases, axon guidance, and developmental biology (Figure 6C). Within the “Neuronal system” pathway, many potassium and calcium ion channel genes were up‐regulated, as has been shown with NGF stimulation of sensory neurons.^3^ Cytoscape network analysis revealed that *Shc1*, *Fgfr1*, and *Plcg1* contribute to all but the “Neuronal system” pathway and represent the genes with the highest degree of connectivity (Figure 6C). *Shc1* and *Plcg1* have been linked to TrkA signaling,^31^ whereas NGF has been shown to induce expression of *Fgfr1*.^32^ All three genes are expressed by nociceptors, with *Fgfr1* additionally expressed by fibroblasts in the DRG, as seen in our scRNAseq database.^17^ Input of the genes contributing to these top five pathways for transcription factor analysis yielded *Prr12*, *Kcnip3*, *Zbtb46*, *Sox10*, and *Kmt2a*. It is of interest that *Sox10* was also one of the top 50 genes regulated by NGF in the DRG (Figure 6B). From our previous scRNAseq data,^17^ *Sox10* is primarily expressed by Schwann cells, and satellite glia in the DRG and has been linked to peripheral nervous system development but not directly to NGF.^33^

Functional enrichment analysis of the down‐regulated genes yielded signal recognition particle‐dependent cotranslational protein targeting to membrane, respiratory electron transport, ATP synthesis by chemiosmotic coupling and heat production by uncoupling proteins, translation initiation complex formation, neutrophil degranulation, and cellular response to chemical stress (Figure 6D). Genes with the highest degree of connectivity for each of the top five down‐regulated pathways include *Rps3*, *Atp5h*, *Rps3*, *Nme2*, and *Cox6b*. None of these genes have been linked to *Ngf* or *Ntrk1*. These results indicate that IA NGF administration activates a transcriptional program marked by up‐regulation of genes related to excitability, neuroplasticity, and sprouting in knee‐innervating DRG.

All animal procedures were approved by the Institutional Animal Care and Use Committee at Rush University Medical Center. The datasets supporting the conclusions of this article are available from the corresponding author on request.

## DISCUSSION

In view of the sizable increase of NGF levels in joints with OA and other arthritides,^9^, ^10^ and given the limited knowledge of NGF's actions on joint tissues despite broad expression of its receptors, we investigated the biologic effects of IA injection of NGF on naïve adult mouse knees. This confirmed previous reports of knee swelling and hyperalgesia following injection of NGF.^16^, ^17^

Although its sensitizing and pain‐producing effects have been reported,^16^, ^34^, ^35^, ^36^ to our knowledge no studies have explored the consequences of long‐term exposure to NGF on individual joint tissues. In all tissues innervated by sensory and sympathetic neurons (such as the skin), NGF production is tightly controlled in basal conditions because alterations of NGF levels can have profound physiologic effects.^2^ Pronounced up‐regulation of NGF during inflammation is expected to have wide‐ranging effects due to the broad expression of both receptors,^11^, ^14^, ^15^, ^37^ as we found to be the case after repeated IA injection of exogenous NGF into naïve knees. First, exposure to NGF caused pathologic changes in the synovium, with increased subintimal cellularity and fibrosis, in addition to lining hyperplasia. In experimental OA, an early increase of synovial NGF is associated with synovitis, as described in the rat monoiodoacetate model^37^ and after rat medial meniscectomy.^38^ Concordant with our findings that NGF‐injected mice developed synovial fibrosis, an older in vitro study reported that NGF induced proliferation of human fibroblast‐like synovial cells.^39^

Synovial pathology induced by NGF was accompanied by robust growth of nociceptors in the medial but not lateral synovium, with neoinnervation in deeper synovial layers. Axonal sprouting is an established biologic effect of NGF and has been reported after endoneurial injection of NGF in rats.^40^ In the skin, ample evidence supports the notion of NGF‐induced nerve growth.^41^ Blockade of NGF using neutralizing antibodies inhibited sprouting of nerve fibers in sarcoma‐injected mice.^42^

Two remarkable observations were noted in the neuronal growth response to NGF. First, newly sprouted synovial fibers had a very similar distribution to the neoinnervation in the synovial sublining that we have reported in OA models,^23^, ^26^ suggesting that neoinnervation in OA may be mediated by NGF. We are planning to address this by evaluating the effect of NGF blockade on nerve sprouting in OA. Second, the growth of nerves in response to NGF was restricted to the medial synovium, with no sprouting in the lateral synovium in this timeframe, which is consistent with our recent findings using 3D lightsheet microscopy.^21^ The growth of preosteophytes was also restricted to the medial compartment even though the whole joint was exposed to NGF. This mirrors what is observed in primary age‐associated OA in mice, in which the medial compartment is also more affected than the lateral,^23^, ^43^ suggesting that the changes in the medial side may be mediated by mechanical loading.

Although chondrocytes express both NGF receptors,^2^, ^11^ exogenous NGF did not cause overt cartilage damage or fibrillation in this timespan. However, longer exposure to NGF might be detrimental to cartilage. The direct effects of NGF on articular cartilage are surprisingly understudied, in spite of the fact that NGF mRNA is up‐regulated in human and murine OA.^13^, ^44^ A recent study reported that NGF exposure increased matrix degradation in OA human cartilage explant,^45^ whereas an anabolic effect of NGF was reported in ATDC5 cells, a chondrocyte cell line.^46^ Although we did not find overt cartilage damage in this time frame, we cannot conclude that there are no biochemical changes in the cartilage. NGF spurred the growth of preosteophytes, with concomitant nociceptor growth in developing osteophytes. NGF also increased subchondral bone in the medial compartment. The role of NGF in bone health is increasingly being studied, both through p75^NTR^ and TrkA signaling. Studies in the DMM model have reported a critical role of p75^NTR^ in bone formation.^47^ A recent study reported that NGF levels acutely increased in a soft tissue injury model, and deletion of *Ngf* or inhibition of TrkA impaired axonal growth into the injured site and reduced trauma‐induced heterotopic bone formation.^48^ Another study demonstrated that exogenous NGF increases bone, which is associated with nerve sprouting.^49^ Taken together, this suggests an important role for NGF‐TrkA signaling in homeostatic bone formation and in heterotopic ossification in response to abnormal conditions. Indeed, cultured osteoblasts can express TrkA receptors and produce NGF, and it has been reported that targeting this axis using a Trk inhibitor induced apoptosis of proliferating osteoblasts, suggesting that autocrine NGF–Trk receptor signaling is essential for the survival of proliferating osteoblasts.^50^ We did not see increased innervation in subchondral bone in response to NGF in this timeframe. Nonetheless, exposure of the joint to exogenous NGF caused marked subchondral bone sclerosis that may reflect the pronounced overall changes in the joint milieu, including inflammation (knee swelling, synovitis) and possibly changes in the articular cartilage in spite of the lack of overt damage, which may contribute to altered loading and changes in the subchondral bone.

To explore molecular mechanisms underpinning the observed biologic effects, we performed scRNAseq on synovia from injected mice. NGF induced a robust multicellular perturbation, particularly within fibroblasts, Schwann cells, and endothelial cells. Synovial lining fibroblasts exhibited the most robust transcriptional response to NGF, with enrichment of pathways (Smo, Wnt) related to fibrosis and matrix deposition, corroborating histologic evidence of lining hyperplasia in response to NGF. These findings are consistent with previous reports that lining fibroblasts express p75^NTR^ and expand in response to NGF signaling.^51^ Lining fibroblasts have also been implicated in osteophyte formation; in response to NGF, these cells were enriched for genes involved in ossification, including up‐regulation of signaling pathways (eg, *Smo*, *Wnt*), transcription factors (eg, *Runx1*), and matrix‐associated factors (eg, *Thbs3*) known to promote endochondral ossification.^52^, ^53^ The synovial lining is contiguous with the periosteum, and lineage‐tracing experiments have previously demonstrated the presence of synovial lining‐derived progenitors within developing osteophytes,^51^ suggesting lining fibroblasts may contribute to osteophyte formation either directly and/or via paracrine signaling with osteochondral lineage cells and that exogenous NGF injection is sufficient to trigger osteophyte formation.

NGF also caused marked alteration of gene expression in knee‐innervating DRG. Although it is known that NGF can regulate gene expression of receptors, transmitters, and ion channels in DRGs,^3^ this is the first report of transcriptome‐wide effects of NGF. Bulk RNAseq supported the neuronal growth promoting effects of NGF, with up‐regulated genes related to axon guidance (*Plxna4*, *Plxnb3*, *Sema5a*) and receptor tyrosine kinase signaling (*Fgfr1*, *Shc1*, *Plcg1*), which are important for axonal growth.^54^ As in the synovium, genes related to Schwann cell function were enriched in the DRG (*Sox10*, *Sema5a*, *Erbb3*).^55^ NGF injection alters the transcriptional program of Schwann cells, activating multiple pathways related to the support of neuron development, including myelination and the regulation of neurotransmitter release/synapse function,^56^, ^57^ suggesting these cells may coordinate with nociceptor afferents to contribute to neuronal sprouting. Schwann cells play an important role in neuronal development and in facilitating peripheral nerve regeneration following injury, but their role in sprouting in response to nonneuronal tissue damage is less studied.^58^ In summary, these experiments confirm the pleiotropic effects of NGF on multiple cell types in the joints, including direct effects on innervating sensory neurons. These effects are likely mediated by direct actions on the neurons after retrograde transport, as well as indirectly through the release of inflammatory mediators from TrkA expressing joint cells, including fibroblasts, chondrocytes, immune cells, as well as sympathetic neurons.

Our study is not without limitations. ScRNAseq analysis of the synovium revealed endothelial pathways in addition to neuronal pathways. Because vascular and neuronal changes often go hand in hand, it would be informative to document the effect of NGF on vascular changes in the joint, but this was beyond the scope of the current study. Furthermore, our study was limited to male mice, whereas sex differences in the response to NGF have been reported. For example, a clinical study demonstrated greater pain intensity and heightened mechanical sensitivity after intramuscular injection of NGF into the masseter muscle of female participants compared with male participants.^59^ Similarly, in a rodent model of low back pain, female rats showed more prolonged mechanical hypersensitivity after intramuscular injection of NGF compared with male rats.^60^ Finally, our study was limited to four weeks, which revealed many rapid onset changes but did not allow us to study the effects of interactions between the different tissues long term.

In summary, the introduction of exogenous NGF into naïve knees resulted in profound structural and neuronal remodeling in multiple joint tissues, mimicking OA‐like changes, including synovial fibrosis, increased subintimal cellularity and hyperplasia of the intima, medial nociceptor sprouting, medial preosteophytes, and increased thickness of medial subchondral bone. Whereas NGF is strongly up‐regulated in OA cartilage,^11^, ^12^, ^13^, ^44^ exogenous IA NGF had no effect on cartilage in this timeframe.

NGF blockade remains an attractive therapeutic strategy for the management of chronic OA pain,^5^ and there are ongoing efforts to target TrkA and p75^NTR^—for example, LEVI‐04, a p75NTR‐Fc—is currently being developed as an inhibitor of neurotrophin 3 (NT3) for OA pain, with promising trial results.^61^ Clinical trials with anti‐NGF antibodies targeted pain in OA and showed analgesic efficacy. However, trials were stopped because of cases of RPOA; these cases occurred in a dose‐dependent manner that is still poorly understood. Although this may appear paradoxical in light of our current findings, several factors should be considered. First, in the current study, NGF was administered locally into the joint cavity, whereas anti‐NGF antibodies were given systemically. Therefore, the target cells for NGF signaling in these two different routes of administration may have been different. Furthermore, we injected NGF into the naïve, healthy knee joints of young animals, whereas anti‐NGF trials targeted patients with moderate to severe knee OA, in which the joint milieu is very different with respect to cellular composition, receptor expression, and innervation. Furthermore, it has been reported that RPOA most commonly occurred in patients treated with tanezumab with more severe radiographic or symptomatic OA.^62^ Finally, it should also be considered that the analgesic effect of anti‐NGF treatment may lead to joint overuse and increased loading of structurally damaged joints, resulting in accelerated damage independent of direct biologic effects.

In conclusion, the picture that arises is a complex and time‐dependent interaction among pain, NGF signaling, nerve growth, and joint damage, and we propose that in‐depth studies with anti‐NGF at different time points in the course of experimental OA are needed. In view of the profound effects of NGF, it will be critical to describe the distribution of NGF and its receptors in all joint tissues in great depth and how they change with age and disease to clarify the differential roles of the receptors and the biologic consequences of blocking NGF signaling in different joint tissues at different stages of the disease.

## AUTHOR CONTRIBUTIONS

All authors contributed to at least one of the following manuscript preparation roles: conceptualization AND/OR methodology, software, investigation, formal analysis, data curation, visualization, and validation AND drafting or reviewing/editing the final draft. As corresponding author, Dr Malfait confirms that all authors have provided the final approval of the version to be published and takes responsibility for the affirmations regarding article submission (eg, not under consideration by another journal), the integrity of the data presented, and the statements regarding compliance with institutional review board/Declaration of Helsinki requirements.

## Supporting information


**Disclosure form**.

**Data S1:** Supplementary Methods

**Supplementary Figure 1:** Schematic illustration of the five experiments performed, showing injection protocols, mouse strains (n), experimental endpoints, and outcome measures. All experiments were conducted using 12‐week old male mice, except for experiment 5, in which 15‐week old male mice were used. ScRNaseq = single cell RNA sequencing. Created with BioRender.com.

**Supplementary Figure 2:** Representative μCT scan of a superior view of the subchondral trabecular bone from the vehicle group showing how the region of interest (ROI) was contoured. Scale bar = 500μm.

**Supplementary Figure 3:** Single cell RNA sequencing (scRNaseq) quality control. (A) Sequencing accuracy quality control measures. (B) Cell quality metrics before and after preprocessing. (C) Spearman correlation of scRNaseq biologic replicates. Each replicate is comprised of synovial cells pooled from ipsilateral synovia of n = 2 mice.

**Supplementary Figure 4:** (A) Knee swelling was assessed in wildtype (WT) mice given repeated IA injections of recombinant murine NGF (50 ng or 500 ng, 5 μL, 2x/week) or vehicle (5 μL, 2x/week) for four weeks; n = 5 mice/group. Red arrowhead indicates the first injection. (B) Knee swelling shown as the area under the curve analysis over the time course. Kruskal‐Wallis test with Dunn's Multiple Comparisons. (C) Knee hyperalgesia was assessed for mice given repeated IA injections of recombinant murine NGF (50 ng or 500 ng, 5 μL, 2x/week) or vehicle (5 μL, 2x/week) for four weeks at 0‐, 2, and 4‐week. Two‐way ANOVA with Dunnett's multiple comparisons test.

**Supplementary Figure 5:** Safranin‐O stained sections of the medial compartment showing chondro‐osteophytes in n = 5 mice/group (vehicle, 50ng NGF and 500ng NGF groups, respectively). Scale bar = 100μm.

**Supplementary Figure 6:** Quantification of Na_V_1.8+ subchondral bone (SCB) channels in the medial compartment of Na_V_1.8‐tdTomato mice injected with vehicle (5 μL, 2x/week) or recombinant murine NGF (500 ng, 5 μL, 2x/week) for four weeks; n = 5 mice/group (A). Mann‐Whitney test. (B,C) Representative confocal images of the medial compartment of vehicle and NGF‐treated groups, respectively. (D) Representative confocal images and (E) phase contrast overlays showing overviews and zoomed in images (in white insets) of medial compartment of NGF‐injected mouse knee. Na_V_1.8+ fibers within growing chondro‐osteophytes are shown in white arrows. Scale bar = 100μm.

**Supplementary Figure 7:** Knee hyperalgesia was assessed at the 4‐week time point for mice given repeated IA injections of recombinant murine NGF (500 ng, 5 μL, 2x/week) or vehicle (5 μL, 2x/week) for four weeks (Experiment 4); this cohort was used for the synovium scRNaseq experiment (Experiment 4); n = 9 mice/group were used to measure knee hyperalgesia, while n = 4 randomly selected mice/group were used for scRNA sequencing. Mann‐Whitney test.

**Supplementary Figure 8:** Counts and clustering of synovial single cell RNA sequencing data. (A) Cell counts per sample following preprocessing to exclude low quality cells and debris. (B) Elbow plot from principal component analysis showing contributions of components 1:30 to overall observed variance. (C) UMAP of synovial cell clustering; (D) Feature plots of established cell type gene markers. (E) Heatmap of top cluster‐defining genes, based on the “FindAllMarkers” function (pct.1 > 0.7, pct.2 < 0.3, sorted by Padj).

**Supplementary Figure 9:** NGF‐induced differential gene expression by cell cluster, shown as volcano plots. Cutoffs at *P*_*adj*_ < 0.05 and |log2(fold change)| > 0.585.

**Supplementary Figure 10:** NGF‐induced gene set enrichment by cell cluster, shown as dot plots of up to top 10 significantly enriched and de‐enriched terms (FDR < 0.05), sorted by normalized enrichment score (NES). Clusters not shown had no significantly enriched terms.

**Supplementary Table 1:** Quality measures for single cell sequencing (derived from 10X Cell Ranger)

**Supplementary Table 2:** Quality measures for DRG bulk RNA sequencing

**Supplementary Table 3:** *P* values for comparisons of knee hyperalgesia between vehicle (control), 50ng NGF (low), and 500ng NGF (high) groups
