# Supplementary material for: Nerve Growth Factor is Sufficient to Cause Multiple Osteoarthritis‐Relevant Pathologic Features in Naïve Murine Knee Joints
Source: Arthritis Rheumatol. 2026 May 10;78(11):2246–60. doi: 10.1002/art.70168 (PMC13712566; doi:10.1002/art.70168)
Supplement: Supplementary file 1 [file ART-78-2246-s001.pdf]

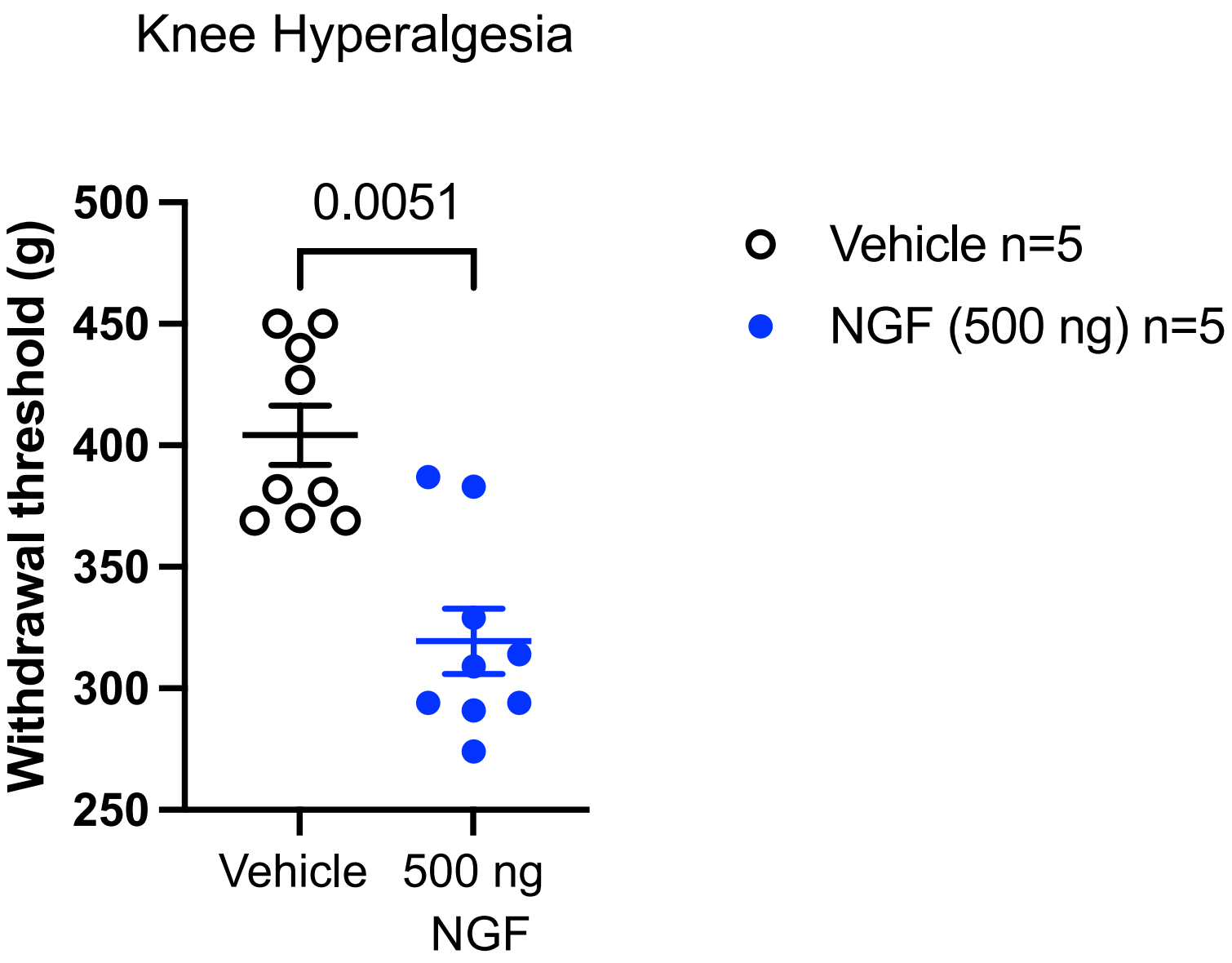
Knee Hyperalgesia
Withdrawal threshold (g)
500
450
400
350
300
250
0.0051
Vehicle
500 ng NGF
Vehicle n=5
NGF (500 ng) n=5
