# Supplementary material for: Nerve Growth Factor is Sufficient to Cause Multiple Osteoarthritis‐Relevant Pathologic Features in Naïve Murine Knee Joints
Source: Arthritis Rheumatol. 2026 May 10;78(11):2246–60. doi: 10.1002/art.70168 (PMC13712566; doi:10.1002/art.70168)
Supplement: Supplementary file 2 [file ART-78-2246-s002.pdf]

ICMJE DISCLOSURE FORM

Date: 7/14/2025
Your Name: Alia Obeidat
Manuscript Title: NERVE GROWTH FACTOR IS SUFFICIENT TO CAUSE MULTIPLE OSTEOARTHRITIS-RELEVANT PATHOLOGICAL FEATURES IN NAÏVE MURINE KNEE JOINTS
Manuscript number (if known): ____

In the interest of transparency, we ask you to disclose all relationships/activities/interests listed below that are related to the content of your manuscript. "Related" means any relation with for-profit or not-for-profit third parties whose interests may be affected by the content of the manuscript. Disclosure represents a commitment to transparency and does not necessarily indicate a bias. If you are in doubt about whether to list a relationship/activity/interest, it is preferable that you do so.

The following questions apply to the author's relationships/activities/interests as they relate to the current manuscript only.

The author's relationships/activities/interests should be defined broadly. For example, if your manuscript pertains to the epidemiology of hypertension, you should declare all relationships with manufacturers of antihypertensive medication, even if that medication is not mentioned in the manuscript.

In item #1 below, report all support for the work reported in this manuscript without time limit. For all other items, the time frame for disclosure is the past 36 months.

		**Name all entities with whom you have this relationship or indicate none (add rows as needed)**	**Specifications/Comments (e.g., if payments were made to you or to your institution)**
		**Time frame: Since the initial planning of the work**	
1	All support for the present manuscript (e.g., funding, provision of study materials, medical writing, article processing charges, etc.) **No time limit for this item.**	___x_None	
		**Time frame: past 36 months**	
2	Grants or contracts from any entity (if not indicated in item #1 above).	___x_None	
3	Royalties or licenses	___x_None	
4	Consulting fees	__x_None	

5	Payment or honoraria for lectures, presentations, speakers bureaus, manuscript writing or educational events	__x__None	
6	Payment for expert testimony	__x__None	
7	Support for attending meetings and/or travel	__x__None	
8	Patents planned, issued or pending	__x__None	
9	Participation on a Data Safety Monitoring Board or Advisory Board	___x_None	
10	Leadership or fiduciary role in other board, society, committee or advocacy group, paid or unpaid	___x_None	
11	Stock or stock options	__x__None	
12	Receipt of equipment, materials, drugs, medical writing, gifts or other services	__x__None	
13	Other financial or non-financial interests	___x_None	

Please place an "X" next to the following statement to indicate your agreement:

_X__ I certify that I have answered every question and have not altered the wording of any of the questions on this form.

ICMJE DISCLOSURE FORM

Date: 7/16/2025
Your Name: Michael Newton
Manuscript Title: NERVE GROWTH FACTOR IS SUFFICIENT TO CAUSE MULTIPLE OSTEOARTHRITIS-RELEVANT PATHOLOGICAL FEATURES IN NAÏVE MURINE KNEE JOINTS
Manuscript number (if known): N/A

In the interest of transparency, we ask you to disclose all relationships/activities/interests listed below that are related to the content of your manuscript. "Related" means any relation with for-profit or not-for-profit third parties whose interests may be affected by the content of the manuscript. Disclosure represents a commitment to transparency and does not necessarily indicate a bias. If you are in doubt about whether to list a relationship/activity/interest, it is preferable that you do so.

The following questions apply to the author's relationships/activities/interests as they relate to the current manuscript only.

The author's relationships/activities/interests should be defined broadly. For example, if your manuscript pertains to the epidemiology of hypertension, you should declare all relationships with manufacturers of antihypertensive medication, even if that medication is not mentioned in the manuscript.

In item #1 below, report all support for the work reported in this manuscript without time limit. For all other items, the time frame for disclosure is the past 36 months.

		**Name all entities with whom you have this relationship or indicate none (add rows as needed)**	**Specifications/Comments (e.g., if payments were made to you or to your institution)**
		**Time frame: Since the initial planning of the work**	
1	All support for the present manuscript (e.g., funding, provision of study materials, medical writing, article processing charges, etc.) **No time limit for this item.**	___x_None	
		**Time frame: past 36 months**	
2	Grants or contracts from any entity (if not indicated in item #1 above).	___x_None	
3	Royalties or licenses	___x_None	
4	Consulting fees	___x_None	

5	Payment or honoraria for lectures, presentations, speakers bureaus, manuscript writing or educational events	___x_None	
6	Payment for expert testimony	___x_None	
7	Support for attending meetings and/or travel	___x_None	
8	Patents planned, issued or pending	___x_None	
9	Participation on a Data Safety Monitoring Board or Advisory Board	___x_None	
10	Leadership or fiduciary role in other board, society, committee or advocacy group, paid or unpaid	___x_None	
11	Stock or stock options	___x_None	
12	Receipt of equipment, materials, drugs, medical writing, gifts or other services	___x_None	
13	Other financial or non-financial interests	___x_None	

Please place an "X" next to the following statement to indicate your agreement:

_X_ I certify that I have answered every question and have not altered the wording of any of the questions on this form.

ICMJE DISCLOSURE FORM

Date: 7/14/2025
Your Name: ____________________Jun Li____
Manuscript Title: NERVE GROWTH FACTOR IS SUFFICIENT TO CAUSE MULTIPLE OSTEOARTHRITIS-RELEVANT PATHOLOGICAL FEATURES IN NAÏVE MURINE KNEE JOINTS
Manuscript number (if known): ____

In the interest of transparency, we ask you to disclose all relationships/activities/interests listed below that are related to the content of your manuscript. "Related" means any relation with for-profit or not-for-profit third parties whose interests may be affected by the content of the manuscript. Disclosure represents a commitment to transparency and does not necessarily indicate a bias. If you are in doubt about whether to list a relationship/activity/interest, it is preferable that you do so.

The following questions apply to the author's relationships/activities/interests as they relate to the current manuscript only.

The author's relationships/activities/interests should be defined broadly. For example, if your manuscript pertains to the epidemiology of hypertension, you should declare all relationships with manufacturers of antihypertensive medication, even if that medication is not mentioned in the manuscript.

In item #1 below, report all support for the work reported in this manuscript without time limit. For all other items, the time frame for disclosure is the past 36 months.

		**Name all entities with whom you have this relationship or indicate none (add rows as needed)**	**Specifications/Comments (e.g., if payments were made to you or to your institution)**
**Time frame: Since the initial planning of the work**			
1	All support for the present manuscript (e.g., funding, provision of study materials, medical writing, article processing charges, etc.) **No time limit for this item.**	_x___None	
**Time frame: past 36 months**			
2	Grants or contracts from any entity (if not indicated in item #1 above).	__x__None	
3	Royalties or licenses	__x__None	
4	Consulting fees	_x___None	

5	Payment or honoraria for lectures, presentations, speakers bureaus, manuscript writing or educational events	__x__None	
6	Payment for expert testimony	__x__None	
7	Support for attending meetings and/or travel	__x__None	
8	Patents planned, issued or pending	_x___None	
9	Participation on a Data Safety Monitoring Board or Advisory Board	__x__None	
10	Leadership or fiduciary role in other board, society, committee or advocacy group, paid or unpaid	__x__None	
11	Stock or stock options	_x___None	
12	Receipt of equipment, materials, drugs, medical writing, gifts or other services	_x___None	
13	Other financial or non-financial interests	__x__None	

Please place an "X" next to the following statement to indicate your agreement:

_x__ I certify that I have answered every question and have not altered the wording of any of the questions on this form.

ICMJE DISCLOSURE FORM

Date: 7/14/2025
Your Name: ______________**Baofeng Hu**____
Manuscript Title: NERVE GROWTH FACTOR IS SUFFICIENT TO CAUSE MULTIPLE OSTEOARTHRITIS-RELEVANT PATHOLOGICAL FEATURES IN NAÏVE MURINE KNEE JOINTS
Manuscript number (if known): ____

In the interest of transparency, we ask you to disclose all relationships/activities/interests listed below that are related to the content of your manuscript. "Related" means any relation with for-profit or not-for-profit third parties whose interests may be affected by the content of the manuscript. Disclosure represents a commitment to transparency and does not necessarily indicate a bias. If you are in doubt about whether to list a relationship/activity/interest, it is preferable that you do so.

The following questions apply to the author's relationships/activities/interests as they relate to the current manuscript only.

The author's relationships/activities/interests should be defined broadly. For example, if your manuscript pertains to the epidemiology of hypertension, you should declare all relationships with manufacturers of antihypertensive medication, even if that medication is not mentioned in the manuscript.

In item #1 below, report all support for the work reported in this manuscript without time limit. For all other items, the time frame for disclosure is the past 36 months.

		**Name all entities with whom you have this relationship or indicate none (add rows as needed)**	**Specifications/Comments (e.g., if payments were made to you or to your institution)**
		**Time frame: Since the initial planning of the work**	
1	All support for the present manuscript (e.g., funding, provision of study materials, medical writing, article processing charges, etc.) **No time limit for this item.**	___x_None	
		**Time frame: past 36 months**	
2	Grants or contracts from any entity (if not indicated in item #1 above).	___x_None	
3	Royalties or licenses	___x_None	
4	Consulting fees	___x_None	

5	Payment or honoraria for lectures, presentations, speakers bureaus, manuscript writing or educational events	___x_None	
6	Payment for expert testimony	___x_None	
7	Support for attending meetings and/or travel	___x_None	
8	Patents planned, issued or pending	___x_None	
9	Participation on a Data Safety Monitoring Board or Advisory Board	___x_None	
10	Leadership or fiduciary role in other board, society, committee or advocacy group, paid or unpaid	___x_None	
11	Stock or stock options	___x_None	
12	Receipt of equipment, materials, drugs, medical writing, gifts or other services	___x_None	
13	Other financial or non-financial interests	___x_None	

Please place an "X" next to the following statement to indicate your agreement:

_x__ I certify that I have answered every question and have not altered the wording of any of the questions on this

Signature: Baofeng Hu

ICMJE DISCLOSURE FORM

Date: 7/14/2025
Your Name: ______________**Shingo Ishihara**____
Manuscript Title: NERVE GROWTH FACTOR IS SUFFICIENT TO CAUSE MULTIPLE OSTEOARTHRITIS-RELEVANT PATHOLOGICAL FEATURES IN NAÏVE MURINE KNEE JOINTS
Manuscript number (if known): ____

In the interest of transparency, we ask you to disclose all relationships/activities/interests listed below that are related to the content of your manuscript. "Related" means any relation with for-profit or not-for-profit third parties whose interests may be affected by the content of the manuscript. Disclosure represents a commitment to transparency and does not necessarily indicate a bias. If you are in doubt about whether to list a relationship/activity/interest, it is preferable that you do so.

The following questions apply to the author's relationships/activities/interests as they relate to the current manuscript only.

The author's relationships/activities/interests should be defined broadly. For example, if your manuscript pertains to the epidemiology of hypertension, you should declare all relationships with manufacturers of antihypertensive medication, even if that medication is not mentioned in the manuscript.

In item #1 below, report all support for the work reported in this manuscript without time limit. For all other items, the time frame for disclosure is the past 36 months.

		**Name all entities with whom you have this relationship or indicate none (add rows as needed)**	**Specifications/Comments (e.g., if payments were made to you or to your institution)**
**Time frame: Since the initial planning of the work**			
1	All support for the present manuscript (e.g., funding, provision of study materials, medical writing, article processing charges, etc.) **No time limit for this item.**	___x_None	
**Time frame: past 36 months**			
2	Grants or contracts from any entity (if not indicated in item #1 above).	___x_None	
3	Royalties or licenses	___x_None	
4	Consulting fees	___x_None	

5	Payment or honoraria for lectures, presentations, speakers bureaus, manuscript writing or educational events	___x_None	
6	Payment for expert testimony	___x_None	
7	Support for attending meetings and/or travel	___x_None	
8	Patents planned, issued or pending	___x_None	
9	Participation on a Data Safety Monitoring Board or Advisory Board	___x_None	
10	Leadership or fiduciary role in other board, society, committee or advocacy group, paid or unpaid	___x_None	
11	Stock or stock options	___x_None	
12	Receipt of equipment, materials, drugs, medical writing, gifts or other services	___x_None	
13	Other financial or non-financial interests	___x_None	

Please place an "X" next to the following statement to indicate your agreement:

_x__ I certify that I have answered every question and have not altered the wording of any of the questions on this

Signature:

ICMJE DISCLOSURE FORM

Date: 7/14/2025
Your Name: Lindsey Lammlin
Manuscript Title: NERVE GROWTH FACTOR IS SUFFICIENT TO CAUSE MULTIPLE OSTEOARTHRITIS-RELEVANT PATHOLOGICAL FEATURES IN NAÏVE MURINE KNEE JOINTS
Manuscript number (if known): ____

In the interest of transparency, we ask you to disclose all relationships/activities/interests listed below that are related to the content of your manuscript. "Related" means any relation with for-profit or not-for-profit third parties whose interests may be affected by the content of the manuscript. Disclosure represents a commitment to transparency and does not necessarily indicate a bias. If you are in doubt about whether to list a relationship/activity/interest, it is preferable that you do so.

The following questions apply to the author's relationships/activities/interests as they relate to the current manuscript only.

The author's relationships/activities/interests should be defined broadly. For example, if your manuscript pertains to the epidemiology of hypertension, you should declare all relationships with manufacturers of antihypertensive medication, even if that medication is not mentioned in the manuscript.

In item #1 below, report all support for the work reported in this manuscript without time limit. For all other items, the time frame for disclosure is the past 36 months.

		**Name all entities with whom you have this relationship or indicate none (add rows as needed)**	**Specifications/Comments (e.g., if payments were made to you or to your institution)**
**Time frame: Since the initial planning of the work**			
1	All support for the present manuscript (e.g., funding, provision of study materials, medical writing, article processing charges, etc.) **No time limit for this item.**	___x_None	
**Time frame: past 36 months**			
2	Grants or contracts from any entity (if not indicated in item #1 above).	___x_None	
3	Royalties or licenses	___x_None	
4	Consulting fees	__x_None	

5	Payment or honoraria for lectures, presentations, speakers bureaus, manuscript writing or educational events	__x__None	
6	Payment for expert testimony	__x__None	
7	Support for attending meetings and/or travel	__x__None	
8	Patents planned, issued or pending	__x__None	
9	Participation on a Data Safety Monitoring Board or Advisory Board	___x_None	
10	Leadership or fiduciary role in other board, society, committee or advocacy group, paid or unpaid	___x_None	
11	Stock or stock options	__x__None	
12	Receipt of equipment, materials, drugs, medical writing, gifts or other services	__x__None	
13	Other financial or non-financial interests	___x_None	

Please place an "X" next to the following statement to indicate your agreement:

_X__ I certify that I have answered every question and have not altered the wording of any of the questions on this form.

ICMJE DISCLOSURE FORM

Date: 7/14/2025
Your Name: Lucas Junginger
Manuscript Title: NERVE GROWTH FACTOR IS SUFFICIENT TO CAUSE MULTIPLE OSTEOARTHRITIS-RELEVANT PATHOLOGICAL FEATURES IN NAÏVE MURINE KNEE JOINTS
Manuscript number (if known): ____

In the interest of transparency, we ask you to disclose all relationships/activities/interests listed below that are related to the content of your manuscript. "Related" means any relation with for-profit or not-for-profit third parties whose interests may be affected by the content of the manuscript. Disclosure represents a commitment to transparency and does not necessarily indicate a bias. If you are in doubt about whether to list a relationship/activity/interest, it is preferable that you do so.

The following questions apply to the author's relationships/activities/interests as they relate to the current manuscript only.

The author's relationships/activities/interests should be defined broadly. For example, if your manuscript pertains to the epidemiology of hypertension, you should declare all relationships with manufacturers of antihypertensive medication, even if that medication is not mentioned in the manuscript.

In item #1 below, report all support for the work reported in this manuscript without time limit. For all other items, the time frame for disclosure is the past 36 months.

		**Name all entities with whom you have this relationship or indicate none (add rows as needed)**	**Specifications/Comments (e.g., if payments were made to you or to your institution)**
		**Time frame: Since the initial planning of the work**	
1	All support for the present manuscript (e.g., funding, provision of study materials, medical writing, article processing charges, etc.) **No time limit for this item.**	__X__None	
		**Time frame: past 36 months**	
2	Grants or contracts from any entity (if not indicated in item #1 above).	__X__None	
3	Royalties or licenses	__X__None	
4	Consulting fees	__X__None	

5	Payment or honoraria for lectures, presentations, speakers bureaus, manuscript writing or educational events	__X__None	
6	Payment for expert testimony	__X__None	
7	Support for attending meetings and/or travel	__X__None	
8	Patents planned, issued or pending	__X__None	
9	Participation on a Data Safety Monitoring Board or Advisory Board	__X__None	
10	Leadership or fiduciary role in other board, society, committee or advocacy group, paid or unpaid	__X__None	
11	Stock or stock options	__X__None	
12	Receipt of equipment, materials, drugs, medical writing, gifts or other services	__X__None	
13	Other financial or non-financial interests	__X__None	

Please place an "X" next to the following statement to indicate your agreement:

__X_ I certify that I have answered every question and have not altered the wording of any of the questions on this form.

ICMJE DISCLOSURE FORM

Date: 7/14/2025
Your Name: ______________________________________Easton Farrell_______________________________________
Manuscript Title: NERVE GROWTH FACTOR IS SUFFICIENT TO CAUSE MULTIPLE OSTEOARTHRITIS-RELEVANT PATHOLOGICAL FEATURES IN NAÏVE MURINE KNEE JOINTS
Manuscript number (if known): ____

In the interest of transparency, we ask you to disclose all relationships/activities/interests listed below that are related to the content of your manuscript. "Related" means any relation with for-profit or not-for-profit third parties whose interests may be affected by the content of the manuscript. Disclosure represents a commitment to transparency and does not necessarily indicate a bias. If you are in doubt about whether to list a relationship/activity/interest, it is preferable that you do so.

The following questions apply to the author's relationships/activities/interests as they relate to the <u>current manuscript only</u>.

The author's relationships/activities/interests should be <u>defined broadly</u>. For example, if your manuscript pertains to the epidemiology of hypertension, you should declare all relationships with manufacturers of antihypertensive medication, even if that medication is not mentioned in the manuscript.

In item #1 below, report all support for the work reported in this manuscript without time limit. For all other items, the time frame for disclosure is the past 36 months.

		**Name all entities with whom you have this relationship or indicate none (add rows as needed)**	**Specifications/Comments (e.g., if payments were made to you or to your institution)**
		**Time frame: Since the initial planning of the work**	
1	All support for the present manuscript (e.g., funding, provision of study materials, medical writing, article processing charges, etc.) **No time limit for this item.**	__X__None	
		**Time frame: past 36 months**	
2	Grants or contracts from any entity (if not indicated in item #1 above).	__X__None	
3	Royalties or licenses	__X__None	
4	Consulting fees	_X___None	

5	Payment or honoraria for lectures, presentations, speakers bureaus, manuscript writing or educational events	__X__None	
6	Payment for expert testimony	___x_None	
7	Support for attending meetings and/or travel	__X__None	
8	Patents planned, issued or pending	__X__None	
9	Participation on a Data Safety Monitoring Board or Advisory Board	__X__None	
10	Leadership or fiduciary role in other board, society, committee or advocacy group, paid or unpaid	_X___None	
11	Stock or stock options	_X___None	
12	Receipt of equipment, materials, drugs, medical writing, gifts or other services	__X__None	
13	Other financial or non-financial interests	__X__None	

Please place an "X" next to the following statement to indicate your agreement:

_X__ I certify that I have answered every question and have not altered the wording of any of the questions on this form.

ICMJE DISCLOSURE FORM

Date: 7/14/2025
Your Name: ___________**Lai Wang**____
Manuscript Title: NERVE GROWTH FACTOR IS SUFFICIENT TO CAUSE MULTIPLE OSTEOARTHRITIS-RELEVANT PATHOLOGICAL FEATURES IN NAÏVE MURINE KNEE JOINTS
Manuscript number (if known): ____

In the interest of transparency, we ask you to disclose all relationships/activities/interests listed below that are related to the content of your manuscript. "Related" means any relation with for-profit or not-for-profit third parties whose interests may be affected by the content of the manuscript. Disclosure represents a commitment to transparency and does not necessarily indicate a bias. If you are in doubt about whether to list a relationship/activity/interest, it is preferable that you do so.

The following questions apply to the author's relationships/activities/interests as they relate to the current manuscript only.

The author's relationships/activities/interests should be defined broadly. For example, if your manuscript pertains to the epidemiology of hypertension, you should declare all relationships with manufacturers of antihypertensive medication, even if that medication is not mentioned in the manuscript.

In item #1 below, report all support for the work reported in this manuscript without time limit. For all other items, the time frame for disclosure is the past 36 months.

		**Name all entities with whom you have this relationship or indicate none (add rows as needed)**	**Specifications/Comments (e.g., if payments were made to you or to your institution)**
**Time frame: Since the initial planning of the work**			
1	All support for the present manuscript (e.g., funding, provision of study materials, medical writing, article processing charges, etc.) **No time limit for this item.**	_X_None	
**Time frame: past 36 months**			
2	Grants or contracts from any entity (if not indicated in item #1 above).	_X_None	
3	Royalties or licenses	_X_None	
4	Consulting fees	_X_None	

5	Payment or honoraria for lectures, presentations, speakers bureaus, manuscript writing or educational events	_X_None	
6	Payment for expert testimony	_X_None	
7	Support for attending meetings and/or travel	_X_None	
8	Patents planned, issued or pending	_X_None	
9	Participation on a Data Safety Monitoring Board or Advisory Board	_X_None	
10	Leadership or fiduciary role in other board, society, committee or advocacy group, paid or unpaid	_X_None	
11	Stock or stock options	_X_None	
12	Receipt of equipment, materials, drugs, medical writing, gifts or other services	_X_None	
13	Other financial or non-financial interests	_X_None	

Please place an "X" next to the following statement to indicate your agreement:

_x__ I certify that I have answered every question and have not altered the wording of any of the questions on this form.

ICMJE DISCLOSURE FORM

Date: 7/14/2025
Your Name: ___**Frank Ko**____
Manuscript Title: NERVE GROWTH FACTOR IS SUFFICIENT TO CAUSE MULTIPLE OSTEOARTHRITIS-RELEVANT PATHOLOGICAL FEATURES IN NAÏVE MURINE KNEE JOINTS
Manuscript number (if known): ____

In the interest of transparency, we ask you to disclose all relationships/activities/interests listed below that are related to the content of your manuscript. "Related" means any relation with for-profit or not-for-profit third parties whose interests may be affected by the content of the manuscript. Disclosure represents a commitment to transparency and does not necessarily indicate a bias. If you are in doubt about whether to list a relationship/activity/interest, it is preferable that you do so.

The following questions apply to the author's relationships/activities/interests as they relate to the current manuscript only.

The author's relationships/activities/interests should be defined broadly. For example, if your manuscript pertains to the epidemiology of hypertension, you should declare all relationships with manufacturers of antihypertensive medication, even if that medication is not mentioned in the manuscript.

In item #1 below, report all support for the work reported in this manuscript without time limit. For all other items, the time frame for disclosure is the past 36 months.

		**Name all entities with whom you have this relationship or indicate none (add rows as needed)**	**Specifications/Comments (e.g., if payments were made to you or to your institution)**
		**Time frame: Since the initial planning of the work**	
1	All support for the present manuscript (e.g., funding, provision of study materials, medical writing, article processing charges, etc.) **No time limit for this item.**	_X_None	
		**Time frame: past 36 months**	
2	Grants or contracts from any entity (if not indicated in item #1 above).	_X_None	
3	Royalties or licenses	_X_None	
4	Consulting fees	_X_None	

5	Payment or honoraria for lectures, presentations, speakers bureaus, manuscript writing or educational events	_X_None	
6	Payment for expert testimony	_X_None	
7	Support for attending meetings and/or travel	_X_None	
8	Patents planned, issued or pending	_X_None	
9	Participation on a Data Safety Monitoring Board or Advisory Board	_X_None	
10	Leadership or fiduciary role in other board, society, committee or advocacy group, paid or unpaid	_X_None	
11	Stock or stock options	_X_None	
12	Receipt of equipment, materials, drugs, medical writing, gifts or other services	_X_None	
13	Other financial or non-financial interests	_X_None	

Please place an "X" next to the following statement to indicate your agreement:

_X_ I certify that I have answered every question and have not altered the wording of any of the questions on this form.

ICMJE DISCLOSURE FORM

Date: 7/14/2025
Your Name: ________**Carla R. Scanzello**____
Manuscript Title: NERVE GROWTH FACTOR IS SUFFICIENT TO CAUSE MULTIPLE OSTEOARTHRITIS-RELEVANT PATHOLOGICAL FEATURES IN NAÏVE MURINE KNEE JOINTS
Manuscript number (if known): ____

In the interest of transparency, we ask you to disclose all relationships/activities/interests listed below that are related to the content of your manuscript. "Related" means any relation with for-profit or not-for-profit third parties whose interests may be affected by the content of the manuscript. Disclosure represents a commitment to transparency and does not necessarily indicate a bias. If you are in doubt about whether to list a relationship/activity/interest, it is preferable that you do so.

The following questions apply to the author's relationships/activities/interests as they relate to the current manuscript only.

The author's relationships/activities/interests should be defined broadly. For example, if your manuscript pertains to the epidemiology of hypertension, you should declare all relationships with manufacturers of antihypertensive medication, even if that medication is not mentioned in the manuscript.

In item #1 below, report all support for the work reported in this manuscript without time limit. For all other items, the time frame for disclosure is the past 36 months.

		**Name all entities with whom you have this relationship or indicate none (add rows as needed)**	**Specifications/Comments (e.g., if payments were made to you or to your institution)**
		**Time frame: Since the initial planning of the work**	
1	All support for the present manuscript (e.g., funding, provision of study materials, medical writing, article processing charges, etc.) **No time limit for this item.**	R01-AR075737 (NIAMS)	Made to institution
		**Time frame: past 36 months**	
2	Grants or contracts from any entity (if not indicated in item #1 above).	____ I01-BX004912 (VA BLR&D PROGRAM)	Made to institution
		R01-AR075737 (NIAMS)	Made to institution
		I01-RX003988 & I50-RX004845 (VA RR&D Program)	Made to institution
3	Royalties or licenses	_X_None	

4	Consulting fees	_X_None	
5	Payment or honoraria for lectures, presentations, speakers bureaus, manuscript writing or educational events	_X_None	
6	Payment for expert testimony	_X_None	
7	Support for attending meetings and/or travel	_X_None	
8	Patents planned, issued or pending	____Patent application # 18/411,242 “COMPOSITIONS AND METHODS FOR TREATING OSTEOARTHRITIS USING A CD14 INHIBITOR”	
9	Participation on a Data Safety Monitoring Board or Advisory Board	_X_None	
10	Leadership or fiduciary role in other board, society, committee or advocacy group, paid or unpaid	Associate Editor, Arthritis & Rheumatology;	
		Editorial Board, Osteoarthritis & Cartilage	
		Editorial Board, Osteoarthritis & Cartilage Open	
11	Stock or stock options	_X_None	
12	Receipt of equipment, materials, drugs, medical writing, gifts or other services	_X_None	
13	Other financial or non-financial interests	_X_None	

Please place an “X” next to the following statement to indicate your agreement:

_X I certify that I have answered every question and have not altered the wording of any of the questions on this form.

ICMJE DISCLOSURE FORM

Date: 7/28/2025
Your Name: ________**Richard J. Miller**____
Manuscript Title: NERVE GROWTH FACTOR IS SUFFICIENT TO CAUSE MULTIPLE OSTEOARTHRITIS-RELEVANT PATHOLOGICAL FEATURES IN NAÏVE MURINE KNEE JOINTS
Manuscript number (if known): ____

In the interest of transparency, we ask you to disclose all relationships/activities/interests listed below that are related to the content of your manuscript. "Related" means any relation with for-profit or not-for-profit third parties whose interests may be affected by the content of the manuscript. Disclosure represents a commitment to transparency and does not necessarily indicate a bias. If you are in doubt about whether to list a relationship/activity/interest, it is preferable that you do so.

The following questions apply to the author's relationships/activities/interests as they relate to the current manuscript only.

The author's relationships/activities/interests should be defined broadly. For example, if your manuscript pertains to the epidemiology of hypertension, you should declare all relationships with manufacturers of antihypertensive medication, even if that medication is not mentioned in the manuscript.

In item #1 below, report all support for the work reported in this manuscript without time limit. For all other items, the time frame for disclosure is the past 36 months.

		**Name all entities with whom you have this relationship or indicate none (add rows as needed)**	**Specifications/Comments (e.g., if payments were made to you or to your institution)**
**Time frame: Since the initial planning of the work**			
1	All support for the present manuscript (e.g., funding, provision of study materials, medical writing, article processing charges, etc.) **No time limit for this item.**	____NIH	Payment to institution
**Time frame: past 36 months**			
2	Grants or contracts from any entity (if not indicated in item #1 above).	_X_None	
3	Royalties or licenses	_X_None	
4	Consulting fees	_X_None	

5	Payment or honoraria for lectures, presentations, speakers bureaus, manuscript writing or educational events	_X_None	
6	Payment for expert testimony	_X_None	
7	Support for attending meetings and/or travel	_X_None	
8	Patents planned, issued or pending	_X_None	
9	Participation on a Data Safety Monitoring Board or Advisory Board	_X_None	
10	Leadership or fiduciary role in other board, society, committee or advocacy group, paid or unpaid	_X_None	
11	Stock or stock options	_X_None	
12	Receipt of equipment, materials, drugs, medical writing, gifts or other services	_X_None	
13	Other financial or non-financial interests	_X_None	

Please place an “X” next to the following statement to indicate your agreement:

__x_ I certify that I have answered every question and have not altered the wording of any of the questions on this form.

ICMJE DISCLOSURE FORM

Date: 7/14/2025
Your Name: _____**Tristan Maerz, PhD**____
Manuscript Title: NERVE GROWTH FACTOR IS SUFFICIENT TO CAUSE MULTIPLE OSTEOARTHRITIS-RELEVANT PATHOLOGICAL FEATURES IN NAÏVE MURINE KNEE JOINTS
Manuscript number (if known): ____

In the interest of transparency, we ask you to disclose all relationships/activities/interests listed below that are related to the content of your manuscript. "Related" means any relation with for-profit or not-for-profit third parties whose interests may be affected by the content of the manuscript. Disclosure represents a commitment to transparency and does not necessarily indicate a bias. If you are in doubt about whether to list a relationship/activity/interest, it is preferable that you do so.

The following questions apply to the author's relationships/activities/interests as they relate to the current manuscript only.

The author's relationships/activities/interests should be defined broadly. For example, if your manuscript pertains to the epidemiology of hypertension, you should declare all relationships with manufacturers of antihypertensive medication, even if that medication is not mentioned in the manuscript.

In item #1 below, report all support for the work reported in this manuscript without time limit. For all other items, the time frame for disclosure is the past 36 months.

		**Name all entities with whom you have this relationship or indicate none (add rows as needed)**	**Specifications/Comments (e.g., if payments were made to you or to your institution)**
		**Time frame: Since the initial planning of the work**	
1	All support for the present manuscript (e.g., funding, provision of study materials, medical writing, article processing charges, etc.) **No time limit for this item.**	NIH	NIH grants: R01AR080035, R21AR076487
		**Time frame: past 36 months**	
2	Grants or contracts from any entity (if not indicated in item #1 above).	_X_None	See above
3	Royalties or licenses	__X__None	
4	Consulting fees	__X__None	

5	Payment or honoraria for lectures, presentations, speakers bureaus, manuscript writing or educational events	__X__None	
6	Payment for expert testimony	__X__None	
7	Support for attending meetings and/or travel	__X__None	
8	Patents planned, issued or pending	__X__None	
9	Participation on a Data Safety Monitoring Board or Advisory Board	__X__None	
10	Leadership or fiduciary role in other board, society, committee or advocacy group, paid or unpaid	__X__None	
11	Stock or stock options	_X___None	
12	Receipt of equipment, materials, drugs, medical writing, gifts or other services	__X__None	
13	Other financial or non-financial interests	_X___None	

Please place an "X" next to the following statement to indicate your agreement:

__X_ I certify that I have answered every question and have not altered the wording of any of the questions on this form.

ICMJE DISCLOSURE FORM

Date: 7/14/2025
Your Name: ________Rachel Miller____
Manuscript Title: NERVE GROWTH FACTOR IS SUFFICIENT TO CAUSE MULTIPLE OSTEOARTHRITIS-RELEVANT PATHOLOGICAL FEATURES IN NAÏVE MURINE KNEE JOINTS
Manuscript number (if known): ____

In the interest of transparency, we ask you to disclose all relationships/activities/interests listed below that are related to the content of your manuscript. "Related" means any relation with for-profit or not-for-profit third parties whose interests may be affected by the content of the manuscript. Disclosure represents a commitment to transparency and does not necessarily indicate a bias. If you are in doubt about whether to list a relationship/activity/interest, it is preferable that you do so.

The following questions apply to the author's relationships/activities/interests as they relate to the current manuscript only.

The author's relationships/activities/interests should be defined broadly. For example, if your manuscript pertains to the epidemiology of hypertension, you should declare all relationships with manufacturers of antihypertensive medication, even if that medication is not mentioned in the manuscript.

In item #1 below, report all support for the work reported in this manuscript without time limit. For all other items, the time frame for disclosure is the past 36 months.

		**Name all entities with whom you have this relationship or indicate none (add rows as needed)**	**Specifications/Comments (e.g., if payments were made to you or to your institution)**
		**Time frame: Since the initial planning of the work**	
1	All support for the present manuscript (e.g., funding, provision of study materials, medical writing, article processing charges, etc.) **No time limit for this item.**	____NIH	Payment to institution
		**Time frame: past 36 months**	
2	Grants or contracts from any entity (if not indicated in item #1 above).	_X___None	
3	Royalties or licenses	_X___None	
4	Consulting fees	_X___None	

5	Payment or honoraria for lectures, presentations, speakers bureaus, manuscript writing or educational events	_X___None	
6	Payment for expert testimony	_X___None	
7	Support for attending meetings and/or travel	_X___None	
8	Patents planned, issued or pending	_X___None	
9	Participation on a Data Safety Monitoring Board or Advisory Board	_X___None	
10	Leadership or fiduciary role in other board, society, committee or advocacy group, paid or unpaid	Associate Editor in Arthritis & Rheumatology	
11	Stock or stock options	_X___None	
12	Receipt of equipment, materials, drugs, medical writing, gifts or other services	_X___None	
13	Other financial or non-financial interests	_X___None	

Please place an "X" next to the following statement to indicate your agreement:

__x_ I certify that I have answered every question and have not altered the wording of any of the questions on this form.

ICMJE DISCLOSURE FORM

Date: 7/28/2025
Your Name: ________Anne-Marie Malfait____
Manuscript Title: NERVE GROWTH FACTOR IS SUFFICIENT TO CAUSE MULTIPLE OSTEOARTHRITIS-RELEVANT PATHOLOGICAL FEATURES IN NAÏVE MURINE KNEE JOINTS
Manuscript number (if known): ____

In the interest of transparency, we ask you to disclose all relationships/activities/interests listed below that are related to the content of your manuscript. "Related" means any relation with for-profit or not-for-profit third parties whose interests may be affected by the content of the manuscript. Disclosure represents a commitment to transparency and does not necessarily indicate a bias. If you are in doubt about whether to list a relationship/activity/interest, it is preferable that you do so.

The following questions apply to the author's relationships/activities/interests as they relate to the current manuscript only.

The author's relationships/activities/interests should be defined broadly. For example, if your manuscript pertains to the epidemiology of hypertension, you should declare all relationships with manufacturers of antihypertensive medication, even if that medication is not mentioned in the manuscript.

In item #1 below, report all support for the work reported in this manuscript without time limit. For all other items, the time frame for disclosure is the past 36 months.

		**Name all entities with whom you have this relationship or indicate none (add rows as needed)**	**Specifications/Comments (e.g., if payments were made to you or to your institution)**
		**Time frame: Since the initial planning of the work**	
1	All support for the present manuscript (e.g., funding, provision of study materials, medical writing, article processing charges, etc.) **No time limit for this item.**	____NIH	Payment to institution
		R01AR064251, R01AR060364, P30AR079206 and UC2AR082186 (NIAMS)	
		**Time frame: past 36 months**	
2	Grants or contracts from any entity (if not indicated in item #1 above).	Orion and Eli Lilly	
3	Royalties or licenses	_X___None	

4	Consulting fees	Roivant, Merck, and Ceva.	
5	Payment or honoraria for lectures, presentations, speakers bureaus, manuscript writing or educational events	_X___None	
6	Payment for expert testimony	_X___None	
7	Support for attending meetings and/or travel	_X___None	
8	Patents planned, issued or pending	_X___None	
9	Participation on a Data Safety Monitoring Board or Advisory Board	_X___None	
10	Leadership or fiduciary role in other board, society, committee or advocacy group, paid or unpaid	Co- Editor-in-Chief, Osteoarthritis and Cartilage	
11	Stock or stock options	_X___None	
12	Receipt of equipment, materials, drugs, medical writing, gifts or other services	_X___None	
13	Other financial or non-financial interests	_X___None	

Please place an "X" next to the following statement to indicate your agreement:

__x_ I certify that I have answered every question and have not altered the wording of any of the questions on this form.
