# Supplementary material for: Nerve Growth Factor is Sufficient to Cause Multiple Osteoarthritis‐Relevant Pathologic Features in Naïve Murine Knee Joints
Source: Arthritis Rheumatol. 2026 May 10;78(11):2246–60. doi: 10.1002/art.70168 (PMC13712566; doi:10.1002/art.70168)
Supplement: Supplementary file 3 [file ART-78-2246-s003.pdf]

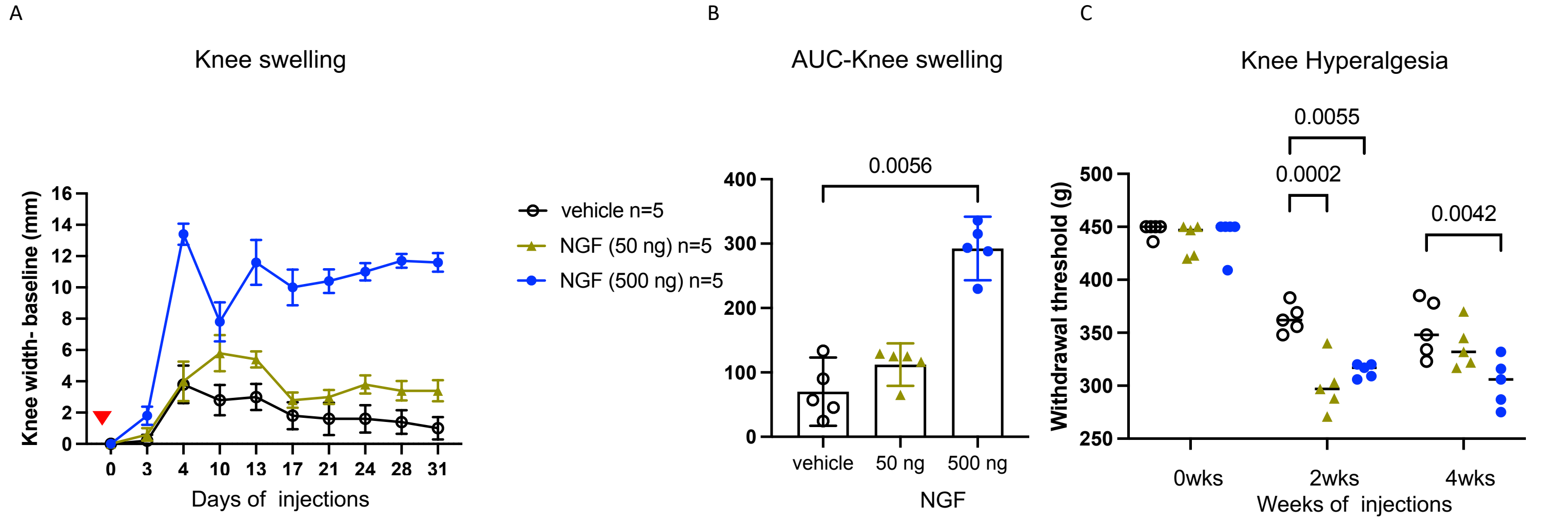
A
Knee swelling
Knee width- baseline (mm)
16
14
12
10
8
6
4
2
0
0
3
4
10
13
17
21
24
28
31
Days of injections
vehicle n=5
NGF (50 ng) n=5
NGF (500 ng) n=5
B
AUC-Knee swelling
0.0056
400
300
200
100
0
vehicle
50 ng
500 ng
NGF
C
Knee Hyperalgesia
0.0055
0.0002
0.0042
Withdrawal threshold (g)
500
450
400
350
300
250
0wks
2wks
4wks
Weeks of injections
