# Supplementary material for: Nerve Growth Factor is Sufficient to Cause Multiple Osteoarthritis‐Relevant Pathologic Features in Naïve Murine Knee Joints
Source: Arthritis Rheumatol. 2026 May 10;78(11):2246–60. doi: 10.1002/art.70168 (PMC13712566; doi:10.1002/art.70168)
Supplement: Supplementary file 4 [file ART-78-2246-s004.pdf]

Cell-Specific Effect of NGF Injection (Volcano Plot, NGF v. Veh)

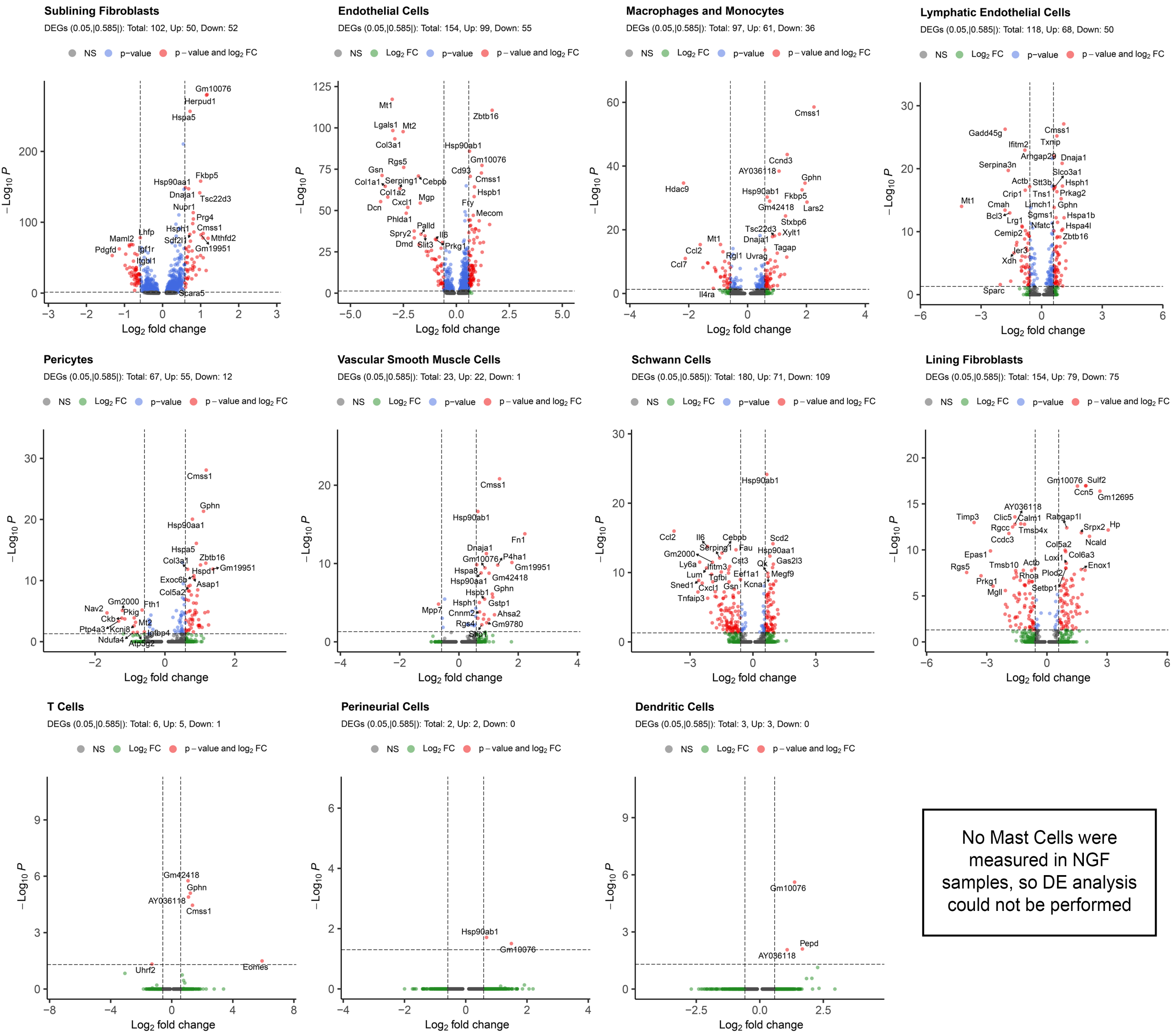

* Differential Expression results were pre-filtered to exclude genes expressed in fewer than 30% of cells within a cluster
