# Supplementary material for: Nerve Growth Factor is Sufficient to Cause Multiple Osteoarthritis‐Relevant Pathologic Features in Naïve Murine Knee Joints
Source: Arthritis Rheumatol. 2026 May 10;78(11):2246–60. doi: 10.1002/art.70168 (PMC13712566; doi:10.1002/art.70168)
Supplement: Supplementary file 5 [file ART-78-2246-s005.pdf]

Comparisons pairs	P-value
+0wks:control vs. +0wks:low	0.9969
+0wks:control vs. +0wks:high	>0.9999
+0wks:control vs. +2wks:control	<0.0001
+0wks:control vs. +2wks:low	<0.0001
+0wks:control vs. +2wks:high	<0.0001
+0wks:control vs. +4wks:control	<0.0001
+0wks:control vs. +4wks:low	<0.0001
+0wks:control vs. +4wks:high	<0.0001
+0wks:low vs. +0wks:high	>0.9999
+0wks:low vs. +2wks:control	<0.0001
+0wks:low vs. +2wks:low	<0.0001
+0wks:low vs. +2wks:high	<0.0001
+0wks:low vs. +4wks:control	<0.0001
+0wks:low vs. +4wks:low	<0.0001
+0wks:low vs. +4wks:high	<0.0001
+0wks:high vs. +2wks:control	<0.0001
+0wks:high vs. +2wks:low	<0.0001
+0wks:high vs. +2wks:high	<0.0001
+0wks:high vs. +4wks:control	<0.0001
+0wks:high vs. +4wks:low	<0.0001
+0wks:high vs. +4wks:high	<0.0001
+2wks:control vs. +2wks:low	0.0002
+2wks:control vs. +2wks:high	0.0055
+2wks:control vs. +4wks:control	0.9946
+2wks:control vs. +4wks:low	0.4134
+2wks:control vs. +4wks:high	0.0004
+2wks:low vs. +2wks:high	0.9444
+2wks:low vs. +4wks:control	0.0018
+2wks:low vs. +4wks:low	0.0708
+2wks:low vs. +4wks:high	>0.9999
+2wks:high vs. +4wks:control	0.0496
+2wks:high vs. +4wks:low	0.6053
+2wks:high vs. +4wks:high	0.9886
+4wks:control vs. +4wks:low	0.8977
+4wks:control vs. +4wks:high	0.0042
+4wks:low vs. +4wks:high	0.1325
