# Supplementary material for: Nerve Growth Factor is Sufficient to Cause Multiple Osteoarthritis‐Relevant Pathologic Features in Naïve Murine Knee Joints
Source: Arthritis Rheumatol. 2026 May 10;78(11):2246–60. doi: 10.1002/art.70168 (PMC13712566; doi:10.1002/art.70168)
Supplement: Supplementary file 6 [file ART-78-2246-s006.pdf]

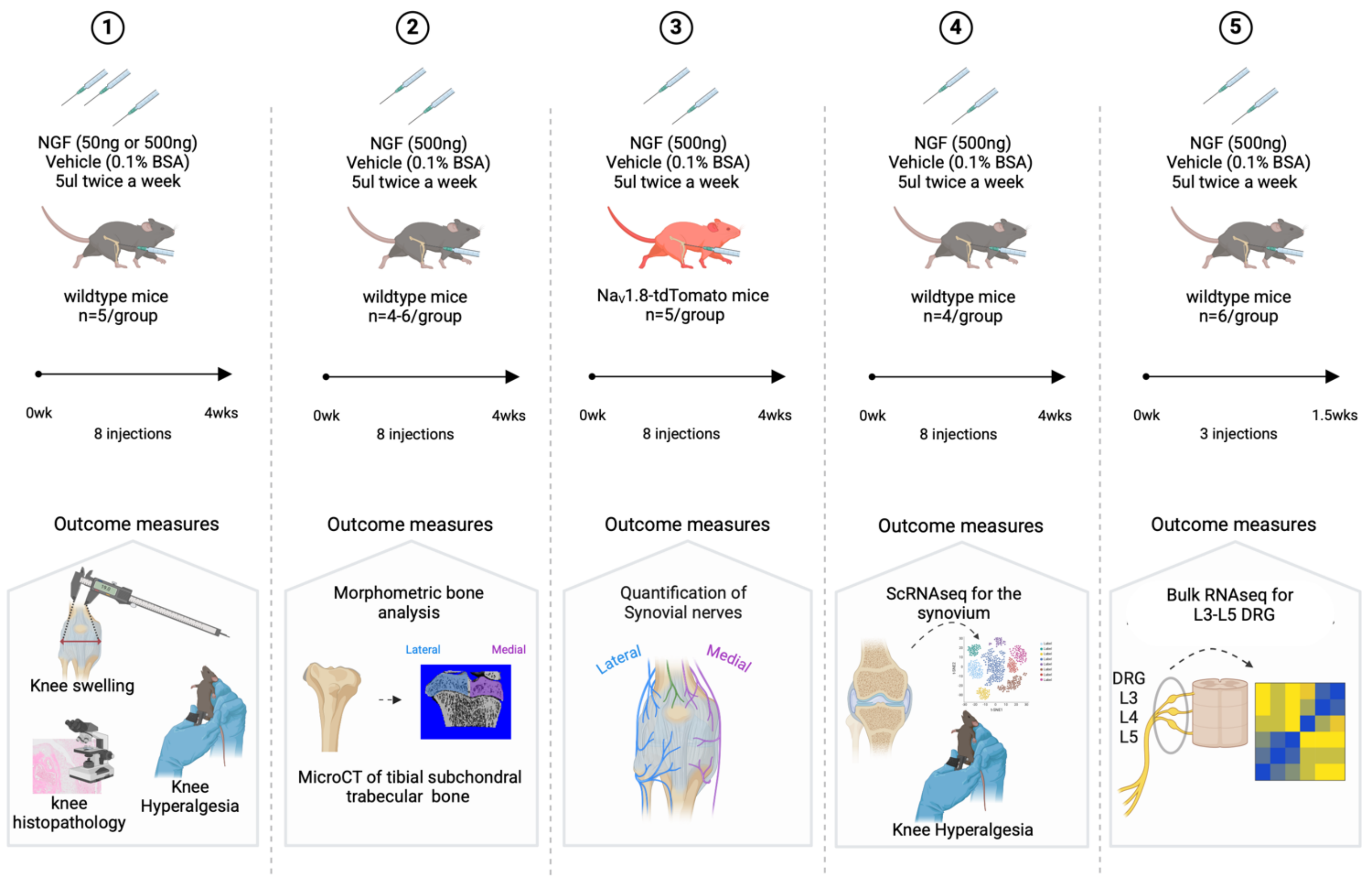
1
NGF (50ng or 500ng)
Vehicle (0.1% BSA)
5ul twice a week
wildtype mice
n=5/group
0wk
4wks
8 injections
Outcome measures
Knee swelling
knee histopathology
Knee Hyperalgesia
2
NGF (500ng)
Vehicle (0.1% BSA)
5ul twice a week
wildtype mice
n=4-6/group
0wk
4wks
8 injections
Outcome measures
Morphometric bone analysis
Lateral
Medial
MicroCT of tibial subchondral trabecular bone
3
NGF (500ng)
Vehicle (0.1% BSA)
5ul twice a week
Na$_V$1.8-tdTomato mice
n=5/group
0wk
4wks
8 injections
Outcome measures
Quantification of Synovial nerves
Lateral
Medial
4
NGF (500ng)
Vehicle (0.1% BSA)
5ul twice a week
wildtype mice
n=4/group
0wk
4wks
8 injections
Outcome measures
ScRNAseq for the synovium
Knee Hyperalgesia
5
NGF (500ng)
Vehicle (0.1% BSA)
5ul twice a week
wildtype mice
n=6/group
0wk
1.5wks
3 injections
Outcome measures
Bulk RNAseq for L3-L5 DRG
DRG
L3
L4
L5
