# Supplementary material for: Nerve Growth Factor is Sufficient to Cause Multiple Osteoarthritis‐Relevant Pathologic Features in Naïve Murine Knee Joints
Source: Arthritis Rheumatol. 2026 May 10;78(11):2246–60. doi: 10.1002/art.70168 (PMC13712566; doi:10.1002/art.70168)

ROI of the μCT scan of the medial and lateral tibial subchondral bone

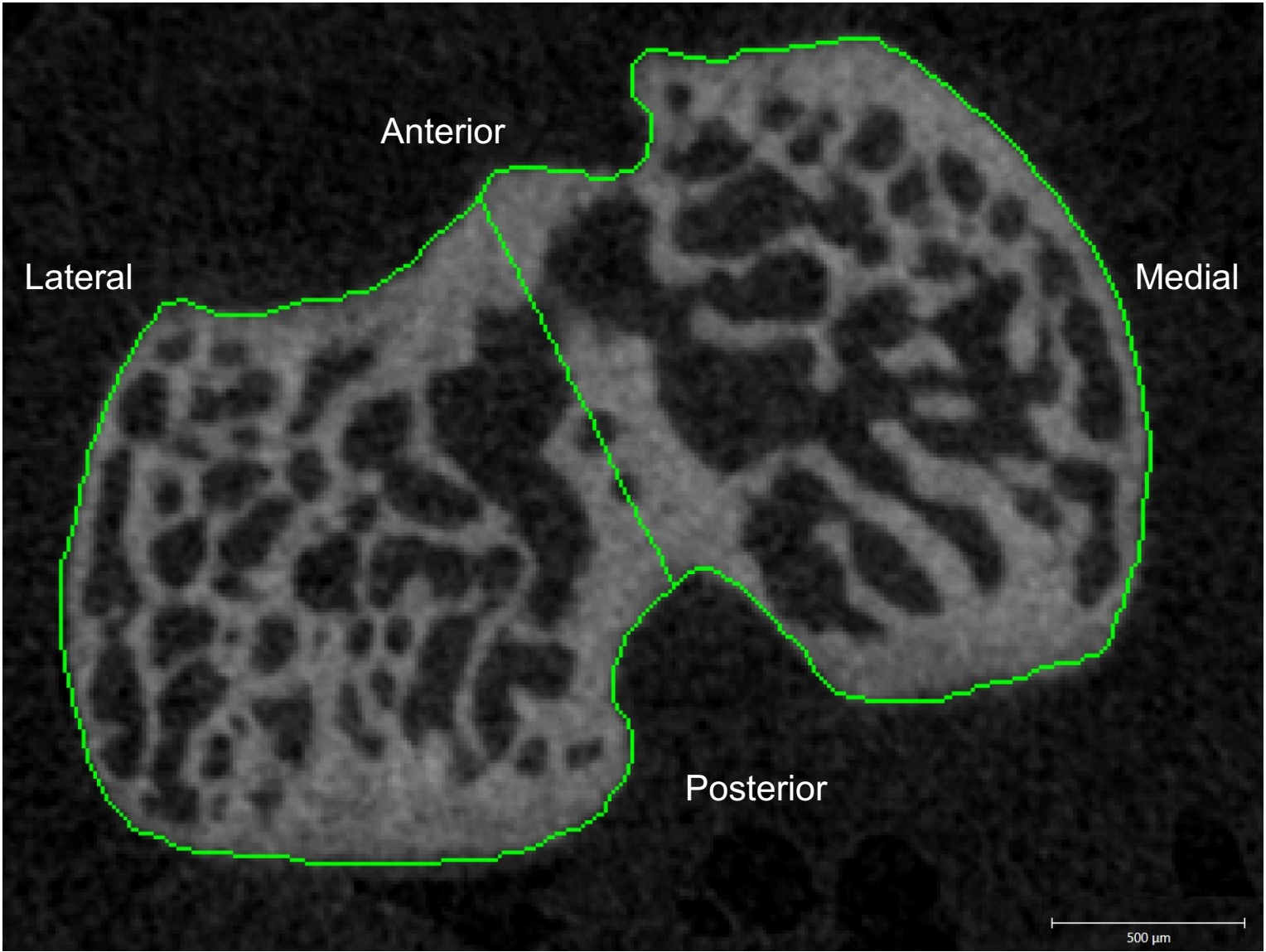

Supplement: Supplementary file 7 [file ART-78-2246-s007.pdf]
