# Supplementary material for: Nerve Growth Factor is Sufficient to Cause Multiple Osteoarthritis‐Relevant Pathologic Features in Naïve Murine Knee Joints
Source: Arthritis Rheumatol. 2026 May 10;78(11):2246–60. doi: 10.1002/art.70168 (PMC13712566; doi:10.1002/art.70168)
Supplement: Supplementary file 8 [file ART-78-2246-s008.pdf]

		Sample			
**Category**	**Quality Measure**	**Veh-1**	**Veh-2**	**NGF-1**	**NGF-2**
Sequencing	Number of Reads	759,282,261	868,920,918	654,938,584	654,736,282
	Number of Short Reads Skipped	0	0	0	0
	Valid Barcodes	97.20%	98.30%	98.20%	98.30%
	Valid UMIs	100.00%	100.00%	100.00%	100.00%
	Sequencing Saturation	82.70%	76.20%	84.60%	79.60%
	Q30 Bases in Barcode	96.00%	96.10%	95.80%	95.80%
	Q30 Bases in RNA Read	91.60%	87.50%	87.40%	87.50%
	Q30 Bases in UMI	95.70%	94.00%	93.30%	93.40%
Cells	Estimated Number of Cells	14,612	42,191	6,694	13,550
	Fraction Reads in Cells	85.20%	78.30%	83.40%	83.10%
	Mean Reads per Cell	51,963	20,595	97,840	48,320
	Median UMI Counts per Cell	2,684	1,155	7,724	2,704
	Median Genes per Cell	1,428	775	2,721	1,368
	Total Genes Detected	25,055	25,091	24,293	24,451
Mapping	Reads Mapped to Genome	92.20%	91.20%	92.80%	90.50%
	Reads Mapped Confidently to Genome	89.60%	88.90%	90.50%	88.10%
	Reads Mapped Confidently to Intergenic Regions	4.80%	6.20%	8.80%	7.50%
	Reads Mapped Confidently to Intronic Regions	13.70%	15.40%	14.10%	13.40%
	Reads Mapped Confidently to Exonic Regions	71.10%	67.30%	67.60%	67.20%
	Reads Mapped Confidently to Transcriptome	80.50%	77.50%	77.50%	76.50%
	Reads Mapped Antisense to Gene	4.00%	4.90%	3.90%	3.90%
