# Supplementary material for: Nerve Growth Factor is Sufficient to Cause Multiple Osteoarthritis‐Relevant Pathologic Features in Naïve Murine Knee Joints
Source: Arthritis Rheumatol. 2026 May 10;78(11):2246–60. doi: 10.1002/art.70168 (PMC13712566; doi:10.1002/art.70168)
Supplement: Supplementary file 9 [file ART-78-2246-s009.pdf]

A

scRNAseq Quality Control Measures - Sequencing Accuracy

	Sample			
Sequencing QC Metric	Veh 1	Veh 2	NGF 1	NGF 2
Number of Reads	7.593E+08	8.689E+08	6.549E+08	6.547E+08
Q30 Bases in UMI	95.7%	94.0%	93.3%	93.4%
Reads Mapped to Genome	92.2%	91.2%	92.8%	90.5%
Reads Mapped Confidently to Genome	89.6%	88.9%	90.5%	88.1%
Reads Mapped Confidently to Exonic Regions	71.1%	67.3%	67.6%	67.2%

B

scRNAseq Quality Control Measures - Cell Quality Preprocessing

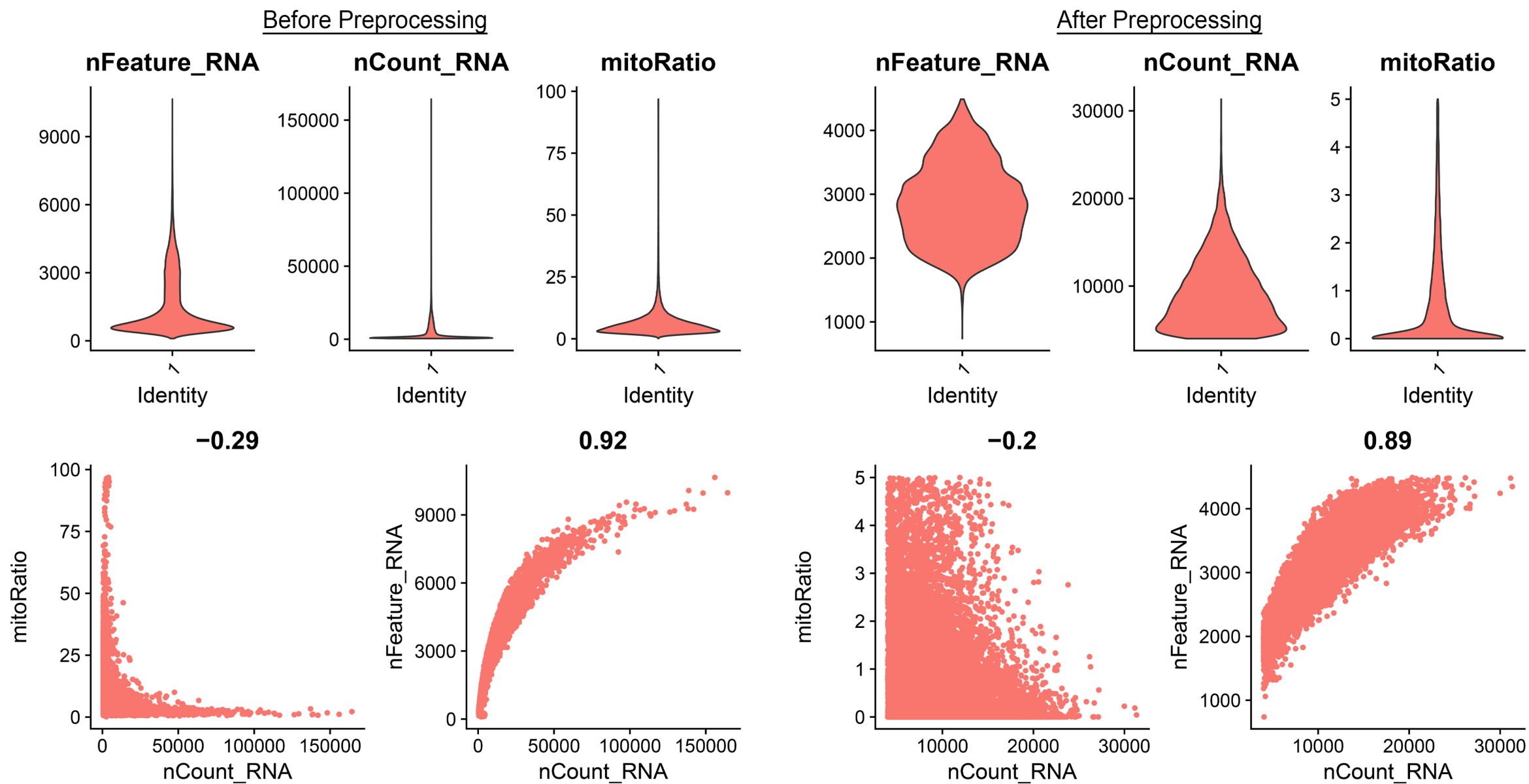

* *Preprocessing includes SoupX removal of ambient RNA, filtering for nCount > 3000, 200 < nFeature < (95th percentile), mitochondrial gene ratio < 5%, and DoubletDetector removal of predicted multiplets.*

C

Spearman Correlation of scRNAseq Bioreps by Cell Cluster

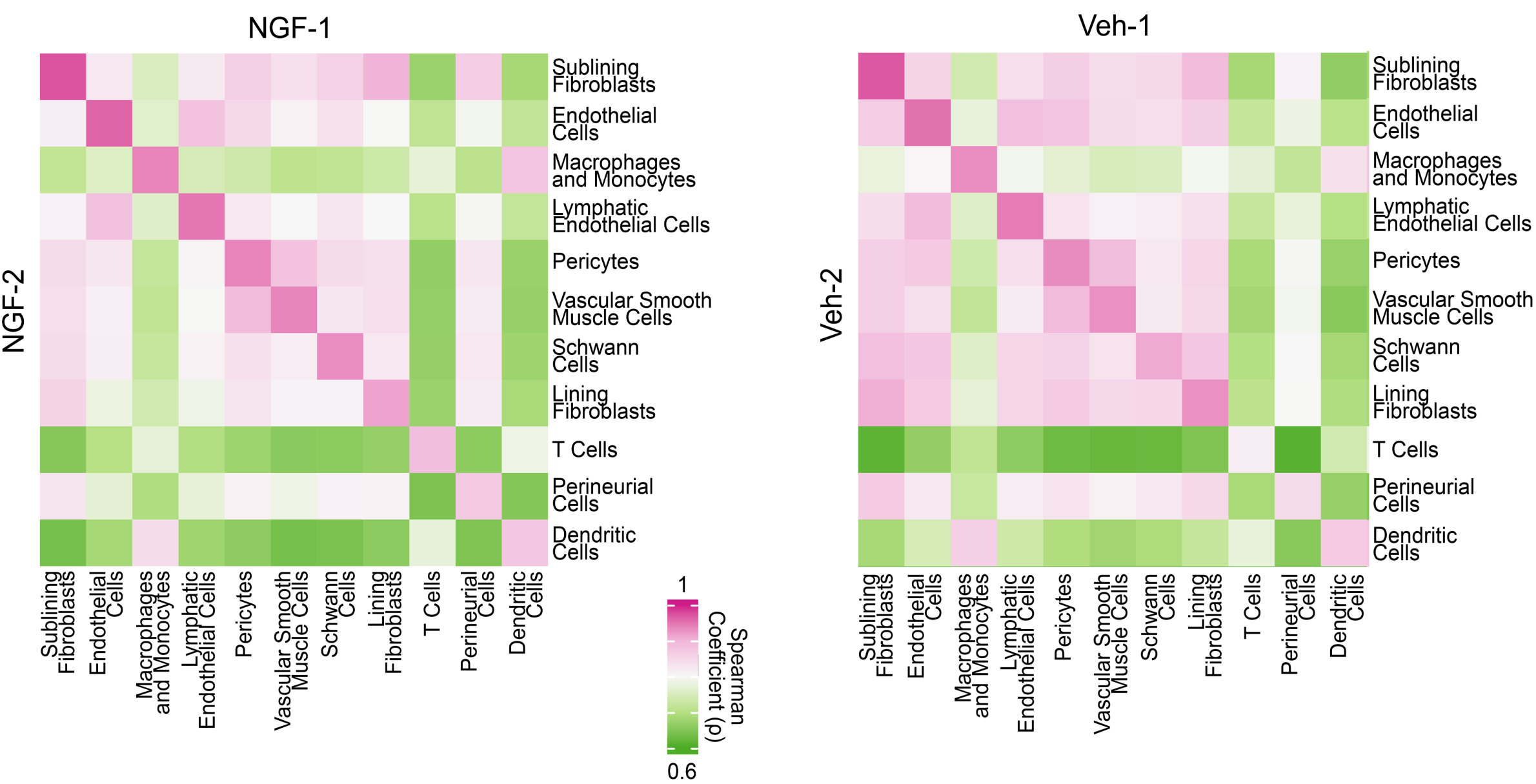

* *Mast Cells not shown in correlation data due to absence from NGF condition and insufficient (n=22) count in Veh-1 for correlation*
