# Supplementary material for: Nerve Growth Factor is Sufficient to Cause Multiple Osteoarthritis‐Relevant Pathologic Features in Naïve Murine Knee Joints
Source: Arthritis Rheumatol. 2026 May 10;78(11):2246–60. doi: 10.1002/art.70168 (PMC13712566; doi:10.1002/art.70168)
Supplement: Supplementary file 10 [file ART-78-2246-s010.pdf]

Sample	Raw Data		Valid Data		Valid Ratio(reads)	Q20%	Q30%	GC content%
	Read	Base	Read	Base				
WN_1	42638654	6.40G	40870934	6.13G	95.85	99.95	96.97	48.50
WN_2	43040698	6.46G	41094752	6.16G	95.48	99.95	96.73	47.50
WN_3	39836506	5.98G	38584682	5.79G	96.86	99.93	97.13	47.50
WN_4	43672604	6.55G	41854180	6.28G	95.84	99.96	97.26	47.50
WN_5	42672090	6.40G	40843672	6.13G	95.72	99.95	97.20	48
WN_6	42644460	6.40G	40845386	6.13G	95.78	99.95	97.19	47.50
WV_1	43050002	6.46G	41826712	6.27G	97.16	99.95	97.19	47.50
WV_2	43716696	6.56G	41840858	6.28G	95.71	99.95	96.88	47.50
WV_3	43331992	6.50G	41468240	6.22G	95.70	99.95	96.87	47.50
WV_4	42035522	6.31G	40212980	6.03G	95.66	99.95	96.95	47.50
WV_5	41457836	6.22G	39621090	5.94G	95.57	99.95	96.80	47.50
WV_6	41434558	6.22G	39533762	5.93G	95.41	99.95	96.86	47.50

Sample	Valid reads	Mapped reads	Unique Mapped reads	Multi Mapped reads	PE Mapped reads	Reads mapped to sense strand	Reads mapped to antisense strand	Non-splice reads	Splice reads
WN_1	40870934	39277412(96.10%)	37783713(92.45%)	1493699(3.65%)	38151880(93.35%)	18908589(46.26%)	18875124(46.18%)	23892360(58.46%)	13891353(33.99%)
WN_2	41094752	39556874(96.26%)	37963415(92.38%)	1593459(3.88%)	38478148(93.63%)	18993986(46.22%)	18969429(46.16%)	23862551(58.07%)	14100864(34.31%)
WN_3	38584682	37313759(96.71%)	35848905(92.91%)	1464854(3.80%)	36484912(94.56%)	17932602(46.48%)	17916303(46.43%)	22504091(58.32%)	13344814(34.59%)
WN_4	41854180	40439299(96.62%)	38636218(92.31%)	1803081(4.31%)	39412812(94.17%)	19327473(46.18%)	19308745(46.13%)	24479869(58.49%)	14156349(33.82%)
WN_5	40843672	39252962(96.11%)	37587557(92.03%)	1665405(4.08%)	38162246(93.43%)	18808537(46.05%)	18779020(45.98%)	23891330(58.49%)	13696227(33.53%)
WN_6	40845386	39281047(96.17%)	37571375(91.98%)	1709672(4.19%)	38165726(93.44%)	18802445(46.03%)	18768930(45.95%)	23716337(58.06%)	13855038(33.92%)
WV_1	41826712	40213173(96.14%)	38350973(91.69%)	1862200(4.45%)	39321384(94.01%)	19188763(45.88%)	19162210(45.81%)	23705752(56.68%)	14645221(35.01%)
WV_2	41840858	40338614(96.41%)	38197688(91.29%)	2140926(5.12%)	39235340(93.77%)	19106950(45.67%)	19090738(45.63%)	23478058(56.11%)	14719630(35.18%)
WV_3	41468240	39898314(96.21%)	38196716(92.11%)	1701598(4.10%)	38748884(93.44%)	19114229(46.09%)	19082487(46.02%)	24033934(57.96%)	14162782(34.15%)
WV_4	40212980	38616770(96.03%)	36674090(91.20%)	1942680(4.83%)	37519064(93.30%)	18348423(45.63%)	18325667(45.57%)	23244262(57.80%)	13429828(33.40%)
WV_5	39621090	37856281(95.55%)	35992293(90.84%)	1863988(4.70%)	36692436(92.61%)	18010045(45.46%)	17982248(45.39%)	23103067(58.31%)	12889226(32.53%)
WV_6	39533762	37867489(95.79%)	36009363(91.09%)	1858126(4.70%)	36730300(92.91%)	18021086(45.58%)	17988277(45.50%)	23047854(58.30%)	12961509(32.79%)
