# Supplementary material for: Nerve Growth Factor is Sufficient to Cause Multiple Osteoarthritis‐Relevant Pathologic Features in Naïve Murine Knee Joints
Source: Arthritis Rheumatol. 2026 May 10;78(11):2246–60. doi: 10.1002/art.70168 (PMC13712566; doi:10.1002/art.70168)
Supplement: Supplementary file 11 [file ART-78-2246-s011.pdf]

Cell-Specific Effect of NGF Injection (Gene Set Enrichment Analysis, NGF v. Veh)

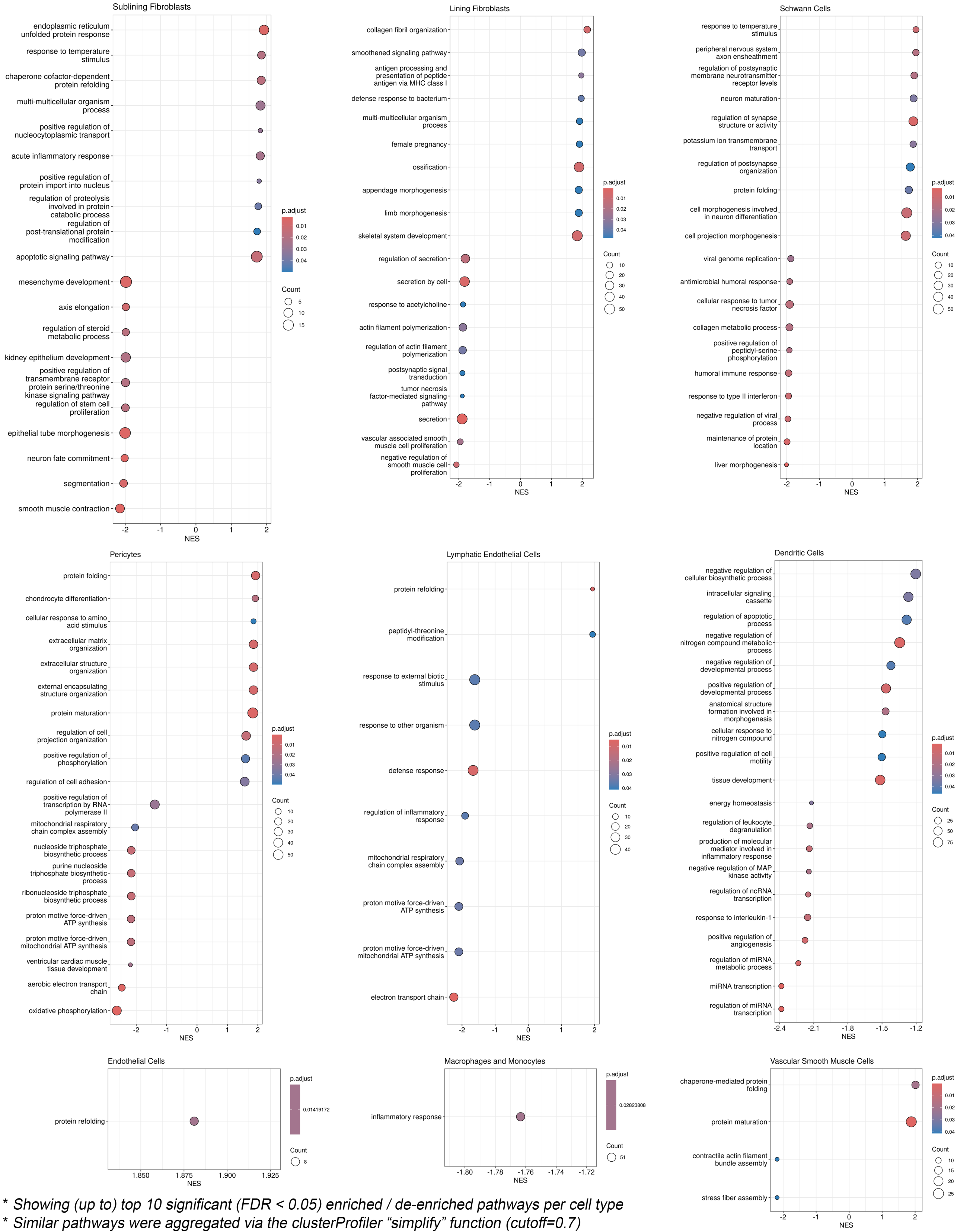

* *Showing (up to) top 10 significant (FDR < 0.05) enriched / de-enriched pathways per cell type*
* *Similar pathways were aggregated via the clusterProfiler "simplify" function (cutoff=0.7)*
