# Supplementary material for: Nerve Growth Factor is Sufficient to Cause Multiple Osteoarthritis‐Relevant Pathologic Features in Naïve Murine Knee Joints
Source: Arthritis Rheumatol. 2026 May 10;78(11):2246–60. doi: 10.1002/art.70168 (PMC13712566; doi:10.1002/art.70168)
Supplement: Supplementary file 13 [file ART-78-2246-s014.pdf]

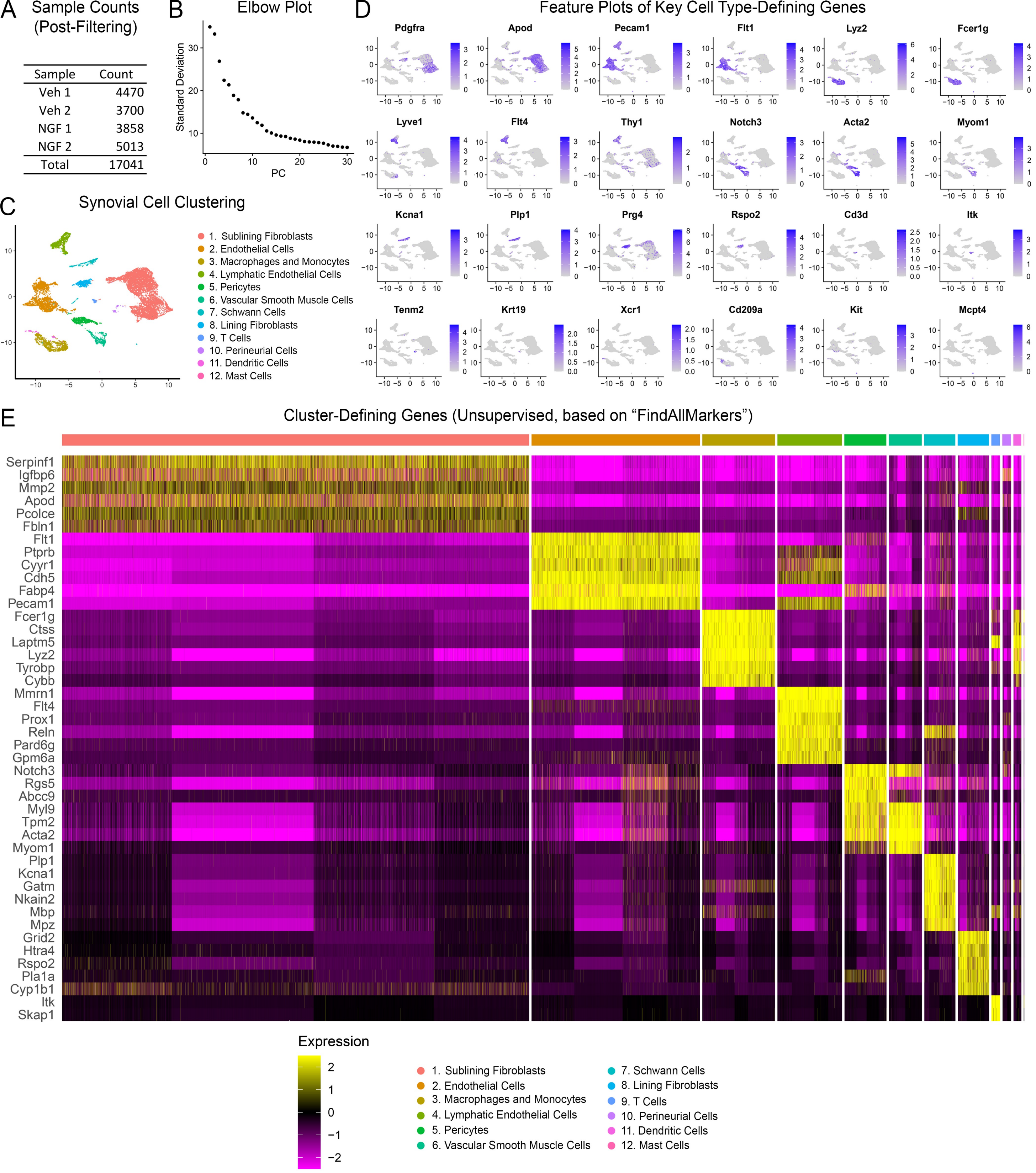


| Sample | Count |
|---|---|
| Veh 1 | 4470 |
| Veh 2 | 3700 |
| NGF 1 | 3858 |
| NGF 2 | 5013 |
| Total | 17041 |
