# Supplementary material for: Nerve Growth Factor is Sufficient to Cause Multiple Osteoarthritis‐Relevant Pathologic Features in Naïve Murine Knee Joints
Source: Arthritis Rheumatol. 2026 May 10;78(11):2246–60. doi: 10.1002/art.70168 (PMC13712566; doi:10.1002/art.70168)
Supplement: Supplementary file 14 [file ART-78-2246-s015.pdf]

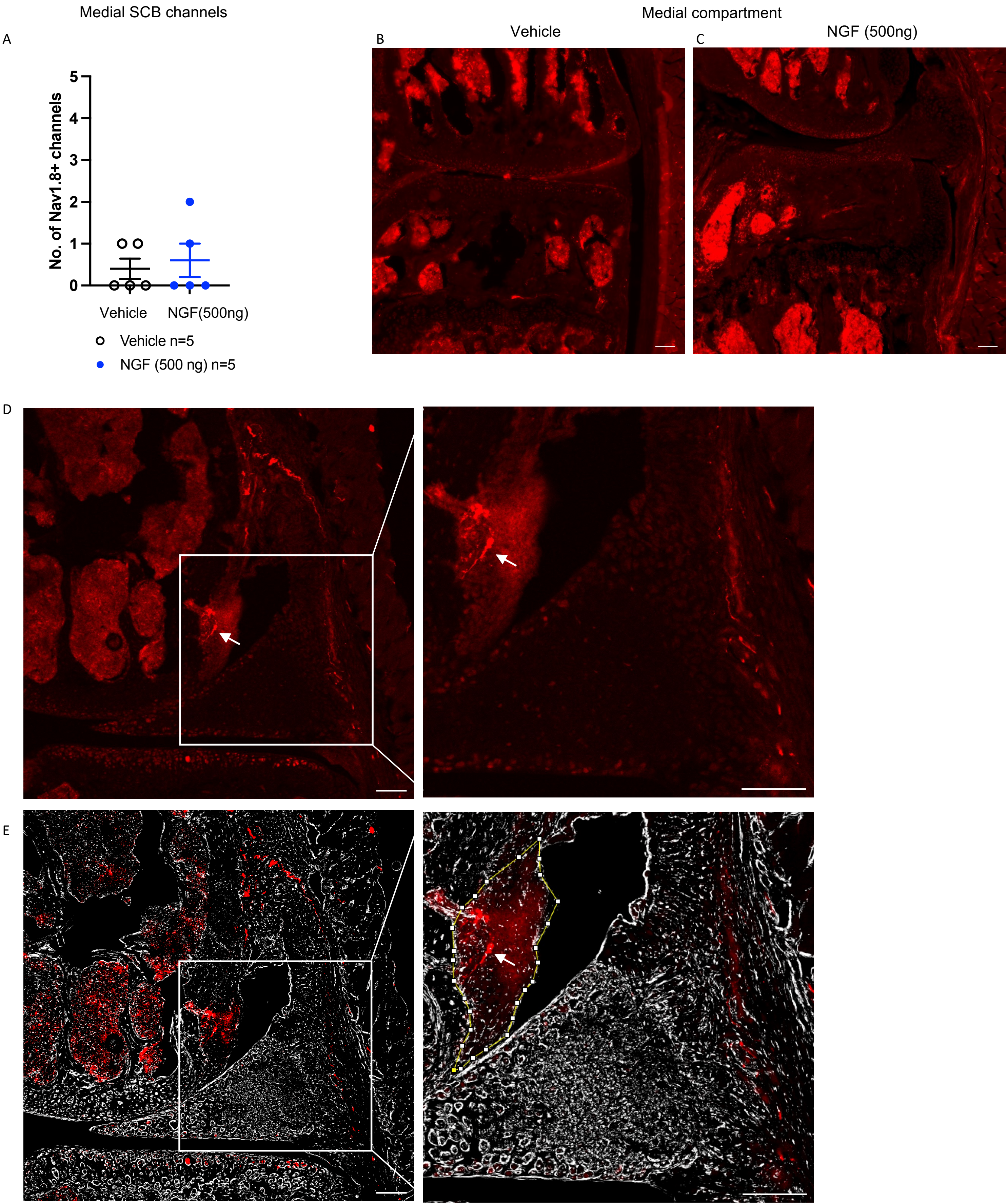

Medial SCB channels
A
No. of Nav1.8+ channels
0
1
2
3
4
5
Vehicle
NGF(500ng)
Vehicle n=5
NGF (500 ng) n=5
Medial compartment
B
Vehicle
C
NGF (500ng)
D
E
