# Supplementary material for: Nerve Growth Factor is Sufficient to Cause Multiple Osteoarthritis‐Relevant Pathologic Features in Naïve Murine Knee Joints
Source: Arthritis Rheumatol. 2026 May 10;78(11):2246–60. doi: 10.1002/art.70168 (PMC13712566; doi:10.1002/art.70168)
Supplement: Supplementary file 15 [file ART-78-2246-s013.docx]

**SUPPLEMENTAL METHODS**

**Knee hyperalgesia**

Knee hyperalgesia was assessed at baseline and then at weeks 2 and 4 using a Pressure Application Measurement (PAM) device as in ^1^. Animals were restrained, and the PAM transducer was pressed against the medial side of the flexed ipsilateral knee. PAM software guided the user to apply a constantly increasing force (30 g/s) up to a maximum of 450 g, and the force at which the mouse tried to withdraw its knee was recorded ^1^. Two measurements were obtained per knee, and the withdrawal thresholds were averaged and reported.

**Knee Histopathology**

Mice in Experiment 1 were euthanized after 4 weeks of injections (at the age of 16 weeks), and the right knees were collected. The knees were fixed in 10% natural buffered formalin, decalcified in EDTA for 3 weeks, embedded in paraffin, and sectioned at 5μm. Sections from the center of the joint were stained with hematoxylin (1x-Sigma HHS160) and eosin (1%-Sigma E4382) (H&E) for evaluation of cartilage damage based on modified Osteoarthritis Research Society International recommendations ^2^.

**Micro Computed Tomography**

Scans were acquired using a 10 μm3 isotropic voxel, 70 kVp and 114 μA peak x-ray tube potential and intensity, 500 ms integration time, and were subjected to Gaussian filtration. The subchondral bone analysis was performed by manually contouring the outline of the medial and lateral tibial epiphysis separately. A lower threshold of 363 mg HA/cm3 was used to evaluate trabecular bone volume fraction (BV/TV, mm^3^/mm^3^), bone mineral density (BMD, mg HA/cm^3^), mean trabecular thickness (Tb.Th, mm), and mean trabecular separation (Tb.Sp, mm).

**Quantification of the neuronal signal in the synovium**

Briefly, regions of interest (ROI) were manually outlined for each section using anatomical landmarks. Thresholds were adjusted for all images similarly to control for background. The area of positive signal within each ROI was measured and normalized to the total area of the ROI; the percentage of positive signal per ROI is reported. Adjustments were made to brightness and contrast to reflect true colors. All images were treated the same in terms of adjustments to brightness and contrast to minimize bias. Na_V_1.8+ signal was quantified in the medial and lateral synovium in mid-joint coronal sections using Image J ^3^. Quantification was performed by an observer blinded to the treatment groups. Na_V_1.8+ subchondral bone channels were quantified as described ^4^.

**Single cell RNA sequencing data analysis methods**

For single cell sequencing, 8 C57Bl/6 mice (n =2/condition, age 12 weeks) were randomized to receive 4 weeks of biweekly intra-articular injections of NGF (500 ng) or vehicle control for 4 weeks. At the end of week 4, mice were humanely euthanized via CO2 asphyxia, and ipsilateral synovia were collected and digested to yield a single cell suspension, as previously described ^5^. Cell suspensions were frozen in liquid nitrogen until the time of submission. Single cell suspensions were submitted to the University of Michigan Advanced Genomics Core for barcoding and library prep via the 10X Genomics processing pipeline (Chromium Next GEM Single Cell 3’ Kit v3.1). Two bioreplicates were submitted per condition, with each bioreplicate pooled from two male ipsilateral synovia. Pooled libraries underwent paired-end sequencing (100bp+100bp) on the DNBseq G400 system (MGI Tech). Raw data were aligned to the mm10-2020-A mouse transcriptome using STAR ^6^ and were preprocessed in Cell Ranger (v7.0.1). Quality control of sequencing indicated high accuracy across all samples (**Suppl. Fig. 2A).**

Analysis of count matrices from Cell Ranger was performed in R (v4.4.1) via the Seurat toolbox ^7^ (v5.1.0). Count matrices were generated using Cell Ranger (v7.0.1), pre-processed using SoupX^8^ to filter out debris, multiplets^9^, and non-viable cells (**Suppl.Fig.2B**) to remove ambient RNA contamination. Briefly, raw and filtered feature matrices from cell ranger were imported into Seurat. The filtered matrix was normalized and scaled, and variable features calculated via SCTransform ^10^ (v0.4.1), dimensional reduction was performed via RunPCA and RunUMAP (dim=1:30), and cells clustered (FindNeighbors, FindClusters) using default settings to provide a rough annotation of distinct cell types, separation of ambient from cell-specific RNA to be tailored to different cell types. A contamination fraction of 10% was specified, ambient RNA was then predicted and corrected for.

SoupX-preprocessed count matrices were then individually loaded into Seurat and pre-filtered. Objects with nCounts < 4000 or nFeature < 200 and were excluded to remove debris, objects with nFeature above the 95th percentile were excluded to remove multiplets, and objects with a mitochondrial gene ratio > 5% were excluded to remove non-viable cells (**Suppl. Fig. 2B)**

To further reduce the presence of multiplets in the dataset, particularly harder to detect homotypic and heterotypic doublets, DoubletFinder ^9^ (v2.0.4) was utilized. Briefly, the dataset was again normalized and scaled, variable features were computed, and the data underwent dimensionality reduction (dim=1:30) and clustered (resolution=0.3). Parameter pK was set by running paramSweep over PCs 1:30 and selecting the pK which produces the maximum BCmetric. The homotypic doublet proportion estimate was automatically determined via the modelHomotypic, specifying an estimated doublet rate of 5%, and finally doubletFinder was used to predict and exclude multiplets using these settings. This yielded a final population of preprocessed cells for each bioreplicate.

Following filtering and doublet exclusion, datasets were normalized, scaled, and variable features were computed using SCTransform ^10^ - this scaling step included regression of mitochondrial ratio and cell cycle gene expression to minimize the effects of these confounding variables on the dataset. Dimensional reduction was performed via RunPCA (PCs=30), and individual samples were integrated into a single Seurat object via RPCA integration (IntegrateLayers). Unsupervised cell type clustering was performed via FindNeighbors and FindClusters to determine distinct cell types. Cluster markers were identified using FindAllMarkers, focusing on significantly enriched markers (*P_adj_* < 0.05, using the Benjamini-Hochberg correction for multiple comparisons) which were expressed in at least 70% of the cluster of interest, and fewer than 30% of clusters in other cell types. Spearman correlation confirmed strong correlation between cell types across bioreps (**Suppl. Fig. 2C**).

Differential expression (DE) analysis between NGF and Veh was performed within each cell cluster via FindMarkers, with min.pct > 0.3 to avoid genes minimally-expressed within a cluster. Mitochondrial and ribosomal genes were excluded from downstream analysis. Significantly differentially-expressed genes (DEGs) were defined as those with *P_adj_* < 0.05 and absolute log2 fold change > 0.585 (e.g. |fold change| > 1.5). DE results within each cluster were functionally annotated using gene-set enrichment analysis (GSEA) via the clusterProfiler ^11^ (v4.12.0) implementation of the fgsea algorithm ^12^ (v1.30.0). A ranked list of genes was submitted to gseGO using the GO biological processes database, with gene rankings calculated as:

$sign\left( {log}_{2}\left( fold change \right) \right)* -{log}_{10}(Pvalue)$	(1)

Unadjusted *P*-values from DE analysis were used to calculate gene rankings to avoid ties. GSEA results with *P_adj_* < 0.05 were defined as significant. From the preliminary list of significantly enriched pathways, an aggregated list was generated using simplify to aggregate overlapping/similar terms (cutoff=0.7), and these results are reported.

Transcription factor binding motif analysis (i.e. regulon analysis) was performed via RcisTarget ^13^ (1.24.0). A list of significant DEGs used as input. Motif rankings (mm9, 500 bp upstream) and annotations (motifAnnotations_mgi) were acquired from Bioconductor. Recovery curve AUC was calculated via calcAUC, with aucMaxRank set to the default value of 5% the total number of rankings in the database. Significantly enriched binding motifs were calculated via addMotifAnnotations (nesThreshold=3), and genes associated with each motif were annotated via addSignificantGenes (method=‘iCisTarget’). Significant motifs (NES > 3) with high confidence, directly annotated transcription factors associated with them were focused on. Predicted transcription factors were screened to determine which were measurable within the cluster of interest, with mean expression > 0.05 read counts/cell and max expression > 25 read counts/cell used as filters.

**Bulk RNAseq of lumbar DRGs**

Ipsilateral DRGs (L3-L5) were collected from male WT mice (age 15 weeks) that received 3 IA injections of 500ng NGF (n=6) or vehicle (n=6) in 5μL over 10 days. DRGs were collected 24 h after the last injection and lysed in Trizol. RNA was extracted using an RNeasy micro kit (Qiagen) and sent to LC Sciences for sequencing. RNA Integrity Numbers all passed QC and were greater than 8.6. A poly(A) RNA sequencing library was prepared following Illumina’s TruSeq-stranded-mRNA sample preparation protocol. Paired-ended sequencing (150 bp) was performed on Illumina’s NovaSeq 6000 sequencing system (LC Sciences). HISAT2 was used to map reads to the Mus musculus reference genome (ftp://ftp.ensembl.org/pub/release-101/fasta/mus_musculus/dna/) ^14^. The mapped reads of each sample were assembled using StringTie with default parameters, and StringTie and ballgown were used to estimate the expression levels of all transcripts and perform expression abundance for mRNAs by calculating FPKM (fragment per kilobase of transcript per million mapped reads) value. The raw sequence data and counts matrices have been submitted to NCBI Gene Expression Omnibus (GEO) with accession number GSE287526. mRNA differential gene expression analysis was performed by R package DESeq2 ^15^ between two different groups (NGF vs. vehicle). The EnhancedVolcano R package was used to create the Volcano plot. Pathway analysis was performed by inputting upregulated or downregulated genes (padj < 0.05) into Cytoscape v3 and using the largest subnetwork of genes for STRING enrichment analysis for Reactome pathways with a redundancy cutoff of 0.5 applied. Transcription factor analysis was performed using the online ChEA3 tool ^16^; the mean score setting was used.

**SUPPLEMENTAL RESULTS**

**Single cell RNA sequencing of the synovium**

Schwann cells were enriched in pathways related to neuronal growth and myelination, with upregulation of *Megf9* and *Gas2I3*^17^, and upregulated heat shock protein pathway genes (*Hsp90aa1*, *Hsp90ab1*) postulated to play a role in neuron development and regulation^18^. Broad heat shock protein pathway activation was observed in sublining fibroblasts, vascular and lymphatic endothelial cells, macrophages and monocytes, pericytes, and VSMCs, suggesting NGF may activate a generalized stress-response. Sublining and lining fibroblasts exhibited enrichment of pro-inflammatory pathways **(Fig.4F)**, with lining fibroblasts enriched in pathways related to innate immunity, marked by upregulation of *Hp* (haptoglobin)^19^ and histocompatibility genes *H2-D1*, *H2-K1*, and *H2-T22*.

Lining fibroblasts in particular exhibited NGF-induced enrichment of several pathways reflective of synovial histopathology, including fibrosis (collagen fibril organization, smoothened signaling pathway) and osteochondral bone formation (ossification, skeletal system development) **(Fig.4F)**. At the gene-level, lining fibroblasts upregulated numerous genes related to fibrosis and endochondral ossification: collagens, including fibrosis-associated *Col3a1*, *Col5a1*, and *Col6a1*, crosslinking enzymes (*Lox*, *Loxl1*), linking glycoproteins (e.g., *Emilin1*), fibrosis- and endochondral ossification-associated signaling pathways including Wnt (*Rspo2*, *Serpinh1*) and Smoothened (*Smo*, *Gli3*, *Gas1*)^20–27^, transcriptional regulators known to drive ECM target genes (*Runx1*, *Tgfbr3*, *Egr2*) and matrix-associated factors and proteases (*Thbs3*, *Ccn1*, *Mmp2*) related to osteogenesis **(Fig.5A)**. At the gene-level, lining fibroblasts upregulated numerous genes related to fibrosis and endochondral ossification: collagens, including fibrosis-associated *Col3a1*, *Col5a1*, and *Col6a1*, crosslinking enzymes (*Lox*, *Loxl1*), linking glycoproteins (e.g., *Emilin1*), fibrosis- and endochondral ossification-associated signaling pathways including Wnt (*Rspo2*, *Serpinh1*) and Smoothened (*Smo*, *Gli3*, *Gas1*)^20–27^, transcriptional regulators known to drive ECM target genes (*Runx1*, *Tgfbr3*, *Egr2*) and matrix-associated factors and proteases (*Thbs3*, *Ccn1*, *Mmp2*) related to osteogenesis **(Fig.5A)**.

**References:**

1.	Ishihara S, Miller RE, Malfait AM. Assessment of Knee Hyperalgesia in Mice using Pressure Application Measurement. Journal of Visual Experimentation. *JoVE*. 2025;in press.

2.	Miller RE, Tran PB, Ishihara S, Larkin J, Malfait AM. Therapeutic effects of an anti-ADAMTS-5 antibody on joint damage and mechanical allodynia in a murine model of osteoarthritis. *Osteoarthritis Cartilage*. 2016;24(2):299-306. doi:10.1016/j.joca.2015.09.005

3.	Obeidat AM, Ishihara S, Li J, et al. Intra-articular sprouting of nociceptors accompanies progressive osteoarthritis: comparative evidence in four murine models. *Front Neuroanat*. 2024;18:1429124. doi:10.3389/fnana.2024.1429124

4.	Obeidat AM, Miller RE, Miller RJ, Malfait AM. The nociceptive innervation of the normal and osteoarthritic mouse knee. *Osteoarthritis Cartilage*. 2019;27(11):1669-1679. doi:10.1016/j.joca.2019.07.012

5.	Knights AJ, Farrell EC, Ellis OM, et al. Synovial fibroblasts assume distinct functional identities and secrete R-spondin 2 in osteoarthritis. *Ann Rheum Dis*. 2023;82(2):272-282. doi:10.1136/ard-2022-222773

6.	Dobin A, Davis CA, Schlesinger F, et al. STAR: ultrafast universal RNA-seq aligner. *Bioinforma Oxf Engl*. 2013;29(1):15-21. doi:10.1093/bioinformatics/bts635

7.	Hao Y, Hao S, Andersen-Nissen E, et al. Integrated analysis of multimodal single-cell data. *Cell*. 2021;184(13):3573-3587.e29. doi:10.1016/j.cell.2021.04.048

8.	Young MD, Behjati S. SoupX removes ambient RNA contamination from droplet-based single-cell RNA sequencing data. *GigaScience*. 2020;9(12):giaa151. doi:10.1093/gigascience/giaa151

9.	McGinnis CS, Murrow LM, Gartner ZJ. DoubletFinder: Doublet Detection in Single-Cell RNA Sequencing Data Using Artificial Nearest Neighbors. *Cell Syst*. 2019;8(4):329-337.e4. doi:10.1016/j.cels.2019.03.003

10.	Hafemeister C, Satija R. Normalization and variance stabilization of single-cell RNA-seq data using regularized negative binomial regression. *Genome Biol*. 2019;20(1):296. doi:10.1186/s13059-019-1874-1

11.	Wu T, Hu E, Xu S, et al. clusterProfiler 4.0: A universal enrichment tool for interpreting omics data. *Innov Camb Mass*. 2021;2(3):100141. doi:10.1016/j.xinn.2021.100141

12.	Korotkevich G, Sukhov V, Budin N, Shpak B, Artyomov MN, Sergushichev A. Fast gene set enrichment analysis. Published online June 20, 2016. doi:10.1101/060012

13.	Aibar S, González-Blas CB, Moerman T, et al. SCENIC: single-cell regulatory network inference and clustering. *Nat Methods*. 2017;14(11):1083-1086. doi:10.1038/nmeth.4463

14.	Kim D, Langmead B, Salzberg SL. HISAT: a fast spliced aligner with low memory requirements. *Nat Methods*. 2015;12(4):357-360. doi:10.1038/nmeth.3317

15.	Love MI, Huber W, Anders S. Moderated estimation of fold change and dispersion for RNA-seq data with DESeq2. *Genome Biol*. 2014;15(12):550. doi:10.1186/s13059-014-0550-8

16.	Keenan AB, Torre D, Lachmann A, et al. ChEA3: transcription factor enrichment analysis by orthogonal omics integration. *Nucleic Acids Res*. 2019;47(W1):W212-W224. doi:10.1093/nar/gkz446

17.	Brandt-Bohne U, Keene DR, White FA, Koch M. MEGF9: a novel transmembrane protein with a strong and developmentally regulated expression in the nervous system. *Biochem J*. 2007;401(2):447-457. doi:10.1042/BJ20060691

18.	Miller DJ, Fort PE. Heat Shock Proteins Regulatory Role in Neurodevelopment. *Front Neurosci*. 2018;12:821. doi:10.3389/fnins.2018.00821

19.	Shen H, Song Y, Colangelo CM, et al. Haptoglobin activates innate immunity to enhance acute transplant rejection in mice. *J Clin Invest*. 2012;122(1):383-387. doi:10.1172/JCI58344

20.	Zhuang Y, Dai J, Wang Y, et al. MiR-338* targeting smoothened to inhibit pulmonary fibrosis by epithelial-mesenchymal transition. *Am J Transl Res*. 2016;8(7):3206-3213.

21.	Li L, Ran J, Li L, Chen G, Zhang S, Wang Y. Gli3 is a novel downstream target of miR‑200a with an anti‑ﬁbrotic role for progression of liver fibrosis in vivo and in vitro. *Mol Med Rep*. 2020;21(4):1861-1871. doi:10.3892/mmr.2020.10997

22.	Chen K, Chen J, Wang L, et al. Parkin ubiquitinates GATA4 and attenuates the GATA4/GAS1 signaling and detrimental effects on diabetic nephropathy. *FASEB J Off Publ Fed Am Soc Exp Biol*. 2020;34(7):8858-8875. doi:10.1096/fj.202000053R

23.	Tong G, Chen X, Lee J, et al. Fibroblast growth factor 18 attenuates liver fibrosis and HSCs activation via the SMO-LATS1-YAP pathway. *Pharmacol Res*. 2022;178:106139. doi:10.1016/j.phrs.2022.106139

24.	Soung DY, Talebian L, Matheny CJ, et al. Runx1 dose-dependently regulates endochondral ossification during skeletal development and fracture healing. *J Bone Miner Res Off J Am Soc Bone Miner Res*. 2012;27(7):1585-1597. doi:10.1002/jbmr.1601

25.	Ohba S. Hedgehog Signaling in Endochondral Ossification. *J Dev Biol*. 2016;4(2):20. doi:10.3390/jdb4020020

26.	Hankenson KD, Hormuzdi SG, Meganck JA, Bornstein P. Mice with a disruption of the thrombospondin 3 gene differ in geometric and biomechanical properties of bone and have accelerated development of the femoral head. *Mol Cell Biol*. 2005;25(13):5599-5606. doi:10.1128/MCB.25.13.5599-5606.2005

27.	Hu L, Chen W, Qian A, Li YP. Wnt/β-catenin signaling components and mechanisms in bone formation, homeostasis, and disease. *Bone Res*. 2024;12(1):39. doi:10.1038/s41413-024-00342-8
